# The Use of Infrared Spectroscopy for the Quantification of Bioactive Compounds in Food: A Review

**DOI:** 10.3390/molecules28073215

**Published:** 2023-04-04

**Authors:** Joel B. Johnson, Kerry B. Walsh, Mani Naiker, Kashif Ameer

**Affiliations:** 1 School of Health, Medical & Applied Science, Central Queensland University, North Rockhampton, QLD 4701, Australia; 2 Institute of Food Science and Nutrition, University of Sargodha, Sargodha 40100, Pakistan; 3 Department of Integrative Food, Bioscience and Biotechnology, Chonnam National University, Gwangju 61186, Republic of Korea; 4 School of Food Science and Biotechnology, Kyungpook National University, Daegu 41566, Republic of Korea

**Keywords:** phenolics, bioactive compounds, infrared spectroscopy, NIR spectroscopy

## Abstract

Infrared spectroscopy (wavelengths ranging from 750–25,000 nm) offers a rapid means of assessing the chemical composition of a wide range of sample types, both for qualitative and quantitative analyses. Its use in the food industry has increased significantly over the past five decades and it is now an accepted analytical technique for the routine analysis of certain analytes. Furthermore, it is commonly used for routine screening and quality control purposes in numerous industry settings, albeit not typically for the analysis of bioactive compounds. Using the Scopus database, a systematic search of literature of the five years between 2016 and 2020 identified 45 studies using near-infrared and 17 studies using mid-infrared spectroscopy for the quantification of bioactive compounds in food products. The most common bioactive compounds assessed were polyphenols, anthocyanins, carotenoids and ascorbic acid. Numerous factors affect the accuracy of the developed model, including the analyte class and concentration, matrix type, instrument geometry, wavelength selection and spectral processing/pre-processing methods. Additionally, only a few studies were validated on independently sourced samples. Nevertheless, the results demonstrate some promise of infrared spectroscopy for the rapid estimation of a wide range of bioactive compounds in food matrices.

## 1. Introduction

### 1.1. Infrared Spectroscopy

Infrared (IR) spectroscopy is a well-established tool in analytical chemistry, offering a non-invasive, non-destructive and rapid means of assessing the chemical composition of a wide range of sample types. For the purposes of analytical spectroscopy, the infrared spectrum can be divided into three main regions: near-infrared (NIR; 750–2500 nm), mid-infrared (MIR; 4000–400 cm^−1^) and far-infrared (400–10 cm^−1^; rarely used in the food analysis sector). Historically, NIRS has been and continues to be utilised more than MIRS in the food industry due to its lower cost, greater penetrative power (i.e., lower absorption by the sample) that allows for more representative sampling [1] and reduced sample preparation times [2]. Wavelengths in this NIR region are absorbed due to the overtone and combination bands of IR-active bonds, rather than their fundamental tones.

Compared to other analytical methods, the main advantages of IR spectroscopy are its speed, relatively low price of the instrument, and the fact that it is typically non-destructive and non-invasive, lowering or eliminating sample preparation time [3,4]. Furthermore, IR spectroscopy is highly sensitive, requires a small amount of sample and allows users to analyse samples from a wide variety of matrix types, including solids, powders, films, gels, liquids and gases [3], and does not produce any waste [5]. Conversely, the challenges involve interpreting spectra from complex mixtures and the need to create and maintain robust calibration models for quantitative analysis [3]. Briefly, a robust model refers to one which can be used year-after-year without losing accuracy over time, or when applied to different population groups (e.g., different varieties, different geographic locations). 

In addition, IR spectroscopy—particularly NIRS—is best suited for the analysis of macroconstituents (usually those present at concentrations of ~0.5% or higher). Below this concentration range, it is difficult to separate out the signal of the analyte from the rest of the spectral peaks. In many cases reporting the detection of analytes at much lower concentrations, it is likely that NIRS is actually detecting a different analyte present at macro-levels—the concentration of which is correlated with the targeted analyte. This is known as a secondary, or surrogate, correlation [4]. In many cases, this correlation may be unavoidable due to both analytes absorbing in similar regions [6]. In other situations, it may be the only way through which IR spectroscopy can be used to estimate the microconstituent concentration. The use of such secondary correlations is acceptable in many cases—as long as the correlation holds true for all samples analysed. Some publications have reported that these correlations may change between different sample populations or harvest years [6], which may explain the poor performance of independent test sets found in some studies using IR spectroscopy for the analysis of microconstituents.

Despite these limitations, the speed and cost-effectiveness of IR spectroscopy have led to its adoption across many sectors of the food industry. This review focuses on the application of IR spectroscopy (both MIR and NIR) for the quantitative assessment of bioactive compounds in foodstuffs. It concludes with a contemporary perspective on the future of IR spectroscopy for the analysis of bioactive compounds in the food industry and highlights key areas where further research is required.

### 1.2. Key Absorbance Peaks in the MIR and NIR Regions

As previously mentioned, one of the major challenges of working with IR spectroscopy is interpreting the spectra. To aid researchers in this process, this section provides some information on the aetiological functional groups responsible for observed peaks at different locations in the MIR and NIR regions. 

**Figure 1:**
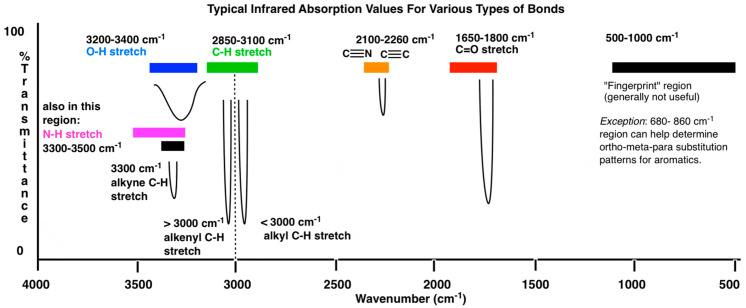
The locations of some major absorption bands in the mid-infrared region. Reproduced from Master Organic Chemistry (https://www.masterorganicchemistry.com/2016/11/23/quick_analysis_of_ir_spectra/) (accessed on 6 January 2022), with kind permission from James Ashenhurst.

**Figure 2:**
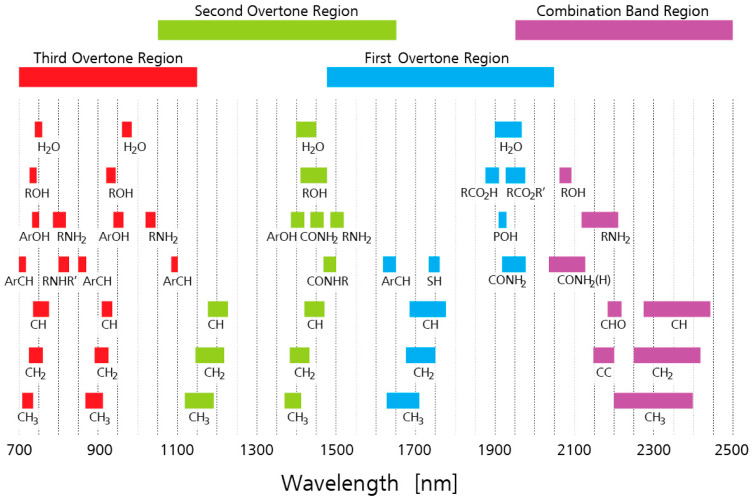
Near-infrared absorption band locations. Reproduced with permission from Metrohm AG, Herisau, Switzerland.

The peak locations of some MIR bonds of particular importance for food analysis are provided in Figure 1 and Table 1. Additionally, the absorption bands in the NIR region are shown in Figure 2. The NIR region contains overtones, meaning that absorption peaks from a single bond occur repeatedly throughout the NIR spectrum, at different levels of attenuation (Figure 2). In addition, combination bands can occur in the far-NIR region (<2000 nm) when two or more fundamental vibrations are excited simultaneously [7]. 

**Table 1:** Mid-infrared absorption bands for a range of bonds important for food analysis.

Bond	Compound/Functional Group	Wavenumbers (cm^−1^) ^1^
O-H stretch	Water, alcohol	3600–3200
C-H stretch	Alkene	3100–3000
C-H stretch	Aromatic ring	3060–3020
C-H stretch	CH_2_/CH_3_	2960–2860
C=O stretch	Carboxylic acid	~1750
C=O stretch	Ester	1750–1715
C=O stretch (amide I)	Amide	1700–1600
C=C stretch	Alkene	1666–1640
C=C stretch	Aromatic ring	1625–1590, 1590–1575, 1525–1470, 1465–1430
-C-H deformation vibration (asymmetric and symmetric)	Methoxy group	1470–1435
O-H deformation	Phenol	1390–1330
C-O-H deformation	Phenol	1382–1317
C-O vibration	Alkyl-aryl ether	1310–1210, 1120–1020
C-O stretch	Phenol	1260–1180
C-C stretch	Phenyl carbon	1225–1075
C-O stretch	Ester, alcohol	1230–1030
-C-H rocking vibration	Methoxy group	1200–1185
C-O stretching vibration	Phenol	1150–1040
C-H out-of-plane deformation	Aromatic ring	900–700
O-H out-of-plane deformation	Aromatic ring	~720

^1^ References: [8,9,10].

### 1.3. Sample Presentation

In order to gain an accurate assessment of the sample matrix using infrared spectroscopy techniques, it is essential that the portion of the sample that the instrument “sees” is representative of the whole sample. Furthermore, due to the wide range of sample types which can be analysed using IR spectroscopy (such as solids, liquids, films, gels and powders), there are a variety of sample presentation methods that have been adopted for IR spectroscopy. 

Perhaps the simplest form of sample presentation is the full transmittance mode (180° light-sample-detector). This is also the only method for which the Beer–Lambert law holds true. In this presentation mode, the IR light enters one side of the sample and some wavelengths are absorbed by the sample, with the remaining light measured as it exits the other side of the sample. As long as the length of the light path is sufficiently low, transmittance mode ensures that the emitted light has an opportunity to interact with nearly all of the analytes present in the light path. Consequently, it is usually quite representative of the true matrix composition. However, it is only suitable for analysing relatively thin samples due to the high level of absorbance in aqueous-based matrices. As shown by Beer–Lambert’s law, increasing the light path length will proportionally increase the absorbance, making it more difficult to detect the signal of the resultant spectra. For example, a path length of only a few millimetres is often required when using transmittance NIR spectroscopy for the analysis of aqueous-based solutions. Due to path length limitations, the use of transmittance spectroscopy for the analysis of solid or powder substances can be more challenging compared to reflectance modes; however, analysis of whole fruits is possible using higher incidence light intensities and more sensitive detectors [11,12]. 

**Figure 3:**
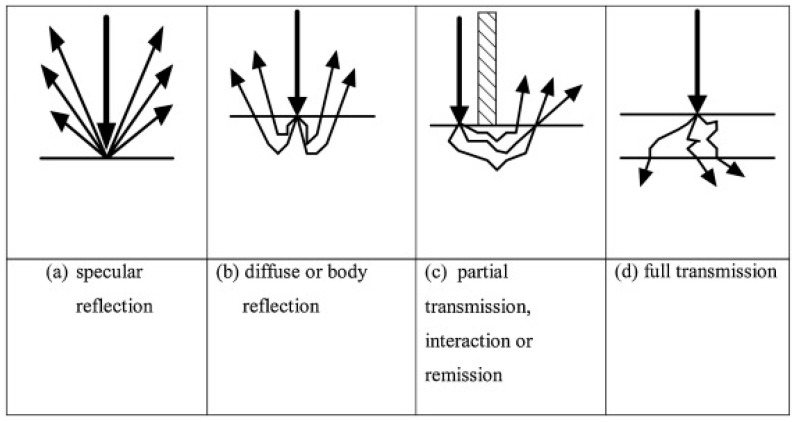
Sample presentation modes used in the infrared spectroscopy analysis of solid materials, showing the interaction of the light with the sample. The arrows indicate representative light paths, with respect to the material being analysed. Reproduced from Walsh et al. [4] under Creative Commons 4.0 licence.

One variation of the full transmittance mode is partial transmission spectroscopy, also known as interactance spectroscopy. This refers to the mode where the infrared light is partially transmitted through the sample matrix, before being detected by another sensor at the matrix surface, but located adjacent to the source. These instruments utilise a physical barrier between the light source and detector to prevent the detector from receiving any IR light reflected from the sample surface (see Figure 3). The benefits of this method are a reduced path length compared to full transmittance mode, and increased interaction between the IR light and the sample matrix compared to reflectance geometry.

Reflectance mode is one of the most commonly used presentation modes in IR spectroscopy applications, particularly for NIRS. In this mode, the infrared light enters one side of the sample and interacts with the sample matrix as it penetrates into the sample. The majority of non-absorbed light is then reflected back to the surface of the sample, where it is detected by the instrument sensor. Some non-absorbance scattering of the IR light can also occur, which can bias the resultant spectra. One of the main advantages of reflectance mode is its one-dimensionality (i.e., the instrument only needs access to the sample surface in one location, as opposed to transmittance spectroscopy where both sides of the sample must be accessible), allowing it to be used in a much broader range of applications compared to transmittance spectroscopy. However, it is reliant on the assumption that the composition of the surface material is representative of the entire sample matrix [4]. 

Within the food sector, reflectance NIR spectroscopy is widely reported in publications for the analysis of horticultural produce [13,14] and in the grains industry [15,16]. There are no commercial instruments designed to use this geometry mode for the analysis of whole fruits, as fruit skin composition (e.g., thickness, starch/fibre content, chlorophyll content) can change in populations from year to year, depending on other variables such as rainfall, amount of sunlight, application of fertiliser, etc. In turn, this variability in skin composition would interfere with the NIR spectra and reduce the robustness of the model, which is designed to only measure the internal composition of the fruit. However, reflectance NIR spectroscopy is commonly used for the analysis of ground grain products in industry/commercial settings, as the surfaces of these samples are generally quite representative of the entire sample.

Diffuse or body reflectance mode is also commonly used by NIR spectroscopists. It functions similarly to regular reflectance spectroscopy, but benefits from increased interaction between the IR light and the sample compared with specular (surface) reflectance modes (Figure 3). 

A diagrammatical summary of the main sample presentation modes used in IR spectroscopy is given in Figure 3. As each sample presentation mode has its drawbacks and benefits [17,18], the optimum method will depend on the sample matrix and intended application.

### 1.4. Data Processing

The final stage in the use of infrared spectroscopy for analytical purposes is the processing of the spectral data. In many cases, the signal of the desired analyte may be obscured by other matrix components present in much higher concentrations, such as water or carbohydrate-based structures. The use of modern mathematical data analysis techniques—termed chemometrics—can aid in uncovering minor analyte signals and developing optimum models for the quantification of the analytes. However, it is important to note that no amount of data analysis or chemometrics can “uncover” an analyte if the signal from the analyte is either not present or too low to be detected by the instrument. The exception to this occurs when a secondary correlation exists between the analyte and a macroconstituent that can be detected by NIRS (see Section 1.1).

#### 1.4.1. Spectral Pre-Processing

Typically, IR spectra are subjected to pre-processing before they can be used for quantitative analytical purposes. The aim of this procedure is to remove spectroscopic artefacts from the measurement process, such as random noise, scatter or baseline drift [19,20]. The effects of these artefacts are particularly detrimental when attempting to analyse complex mixtures or analytes present in very low concentrations [21]. 

A variety of spectral pre-processing methods are available. These include smoothing, multiplicative scatter-correction (MSC), standard normal variate (SNV), normalisation by range (NBR) and the calculation of derivatives [22]. As previous authors have reviewed the range of available spectral pre-processing methods in detail [23,24], only a brief summary of the most commonly pre-processing methods is presented here. 

Standard normal variate (SNV) is a normalisation-based pre-processing method. In this pre-processing method, the mean value of each spectrum is calculated and this constant value is subtracted across the entire spectrum, before the spectrum is divided by the standard deviation of the entire spectrum. 

Calculating the derivative of spectra is another common approach to account for baseline shift or amplitude differences in the spectra. First and second derivatives are the most commonly used. Although higher order derivatives, such as the third derivative, have been successfully used in some applications [25,26,27], there is an accompanying decrease in the signal-to-noise ratio as the derivative order increases [25]. 

Finally, it is important to note that pre-processing methods are often combined. For example, typical pre-processing of spectra for use in analytical spectroscopy could involve calculating the SNV of the spectra, before subsequently calculating the first derivative of the SNV-processed spectra. 

The choice of optimum spectral pre-processing methods is poorly defined and strongly dependent upon both the matrix type and analyte of interest. Furthermore, the need for and choice of pre-processing method may also vary with the sample size of the population [21]. In the absence of definitive guidelines, trial and error is often the best approach when developing new applications for infrared spectroscopy.

#### 1.4.2. Data Analysis Methods

For quantitative applications of IR spectroscopy, regression modelling is among the most commonly used data analysis methods. One of the first chemometric methods applied in quantitative IR spectroscopy applications was multiple linear regression (MLR), which attempts to predict the analyte concentration from the spectral absorbance at several different wavelengths. However, it cannot be used for the analysis of entire spectra, due to the high multicollinearity of the datapoints comprising the spectra. 

Partial least squares (PLS) regression is a derivative of MLR suited to datasets with high levels of multicollinearity, such as infrared spectra [28]. Through a variety of algorithms, the key contributing variables are identified and weighted such that the wavelengths most closely correlated with the analyte concentration have the greatest contribution to the PLS model [28]. PLS regression is widely used for the development of IR spectroscopy models across the food science sector [29,30,31]. 

In recent years, there has also been an emerging interest in machine learning techniques, such as artificial neural networks (ANNs), support vector machine (SVM) and deep learning [32,33,34,35]. These non-linear techniques look for patterns within the data in order to optimise model weighting and extract the desired information from the data. As more datapoints are added to the dataset, the model can update over time in order to provide more accurate prediction results. 

As with spectral pre-processing, the optimum chemometric technique may vary depending on the sample matrix and/or analyte [36,37].

## 2. Bioactive Compounds and Their Significance in Functional Foods

### 2.1. Functional Foods

Recent years have seen an expansion of the “functional food” market—where foods are purchased for their health-benefiting effects, rather than as a source of basic nutrition and energy [38,39,40]. For example, the consumption of juice from Queen Garnet plums has been shown to reduce oxidative stress [41] and reduce the risk of blood clot formation in clinical trials [42], while polyphenolics isolated from chickpeas have been found to provide anti-cancer effects, particularly against colorectal cancer [43]. If high levels of such health-benefiting compounds can be demonstrated in a particular crop, consumers may pay a price premium for such a product, particularly if they are familiar with the concepts of functional foods [44]. For example, Spanish consumers reported that they would pay ~55% extra for resveratrol-enriched wine [45], as this compound has purported benefits for cardiovascular health. This willingness to pay a premium for healthier food has been mirrored in several other studies [46,47,48], albeit with typically lower price premiums reported (e.g., 10–15% higher than the regular price). 

Even if there is not a market for the functional foods in its unprocessed form, such produce also has potential for the development of value-added foods and ingredients [49,50,51], marketed on the basis of their levels of health-benefiting compounds. Examples of foods experiencing a considerable rise in popularity due to their reported health benefits include the so-called ancient grains (such as chia, quinoa, millet and spelt), pulse crops (including mungbeans, chickpeas, faba beans and lentils), as well as numerous other crops [52,53,54]. For instance, the plum variety Queen Garnet was developed and marketed with a sole emphasis on its exceptionally high levels of anthocyanins, which possess antioxidative and anti-thrombotic properties [41,55,56]. Another well-known example is the açaí berry from South America, popularised due to its high anthocyanin content and antioxidant capacity [57].

### 2.2. Definition of Bioactive Compounds

There is no clear literature consensus on the definition of bioactive compounds, with Guaadaoui et al. [58] proposing them to be “compounds which have the capability and the ability to interact with one or more component(s) of living tissue by presenting a wide range of probable effects”. However, from a consumer’s perspective, bioactive compounds are generally regarded as compounds which promote good health or provide health-benefitting effects. This is more similar to the consensus statement from the 23rd Hohenheim Consensus Meeting, which stated that “bioactive compounds are essential and non-essential compounds (e.g., vitamins or polyphenols) that occur in nature, are part of the food chain, and can be shown to have an effect on human health” [59]. Such bioactive compounds may also be referred to as “nutraceuticals” [60], which reflects their presence in the human diet.

### 2.3. Classes of Bioactive Compounds

**Figure 4:**
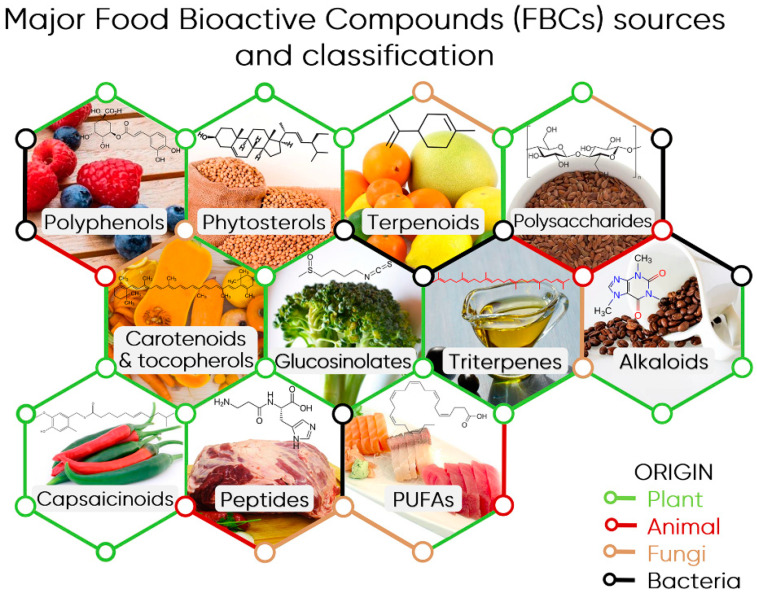
The various classes of major bioactive compounds found in food products. Reproduced from Câmara et al. [63] under Creative Commons 4.0 licence.

The majority of bioactive compounds can be broadly classified as phytochemicals—compounds that are produced by plants—although some (such as fatty acids) are also found in animal-based foods. There are numerous classes of bioactive compounds (Figure 4), each with their own distinct biological activities and health benefits. These include polyphenols, flavonoids, carotenoids, phytosterols, phytoestrogens, alkaloids, glucosinolates, anthocyanins, terpenoids and others [61,62]. Each compound class is characterised by distinct structural features in their chemical composition. For example, polyphenols display the presence of multiple phenol groups, while all flavonoids comprise two phenyl rings and a heterocyclic ring containing an embedded oxygen heteroatom.

It could be considered that compounds which show antioxidant activity form a class of bioactive compounds. However, a structurally diverse array of compounds may exhibit antioxidant activity, including polyphenols, anthocyanins, flavonoids and carotenoids. For this reason, this review excluded studies solely reporting quantification of the total antioxidant capacity (TAC) of samples, as, in many cases, the antioxidant activity of a matrix cannot be directly related to the concentration of a specific structural class of bioactive compounds [64]. Nevertheless, this does not detract from the importance of TAC as a potential indicator of crude biological activity. Although, the concept of TAC has been criticised by several researchers as a result of its lack of specificity [65,66], numerous clinical trials have indicated a positive relationship between a greater intake of antioxidants and reduced levels of oxidative stress and inflammatory markers [67,68,69,70], reduced all-cause mortality (in non-elderly cohorts) [71,72] and reduced risk of adverse cardiovascular events, particularly ischaemic stroke [73,74,75,76,77].

### 2.4. Current Analytical Methods

There are numerous analytical methods available for the quantification of bioactive compounds, depending on the physical and chemical properties of the specific class of compound(s) of interest. 

For example, terpenoids and other volatile compounds are commonly analysed by gas chromatography coupled with mass spectrometry (GC-MS), which uses a mobile inert gas phase and a stationary column phase to separate the compounds of interest [78,79]. 

Non-volatile compounds, such as polyphenols, anthocyanins, flavonoids and carotenoids, are typically analysed using the related technique of liquid chromatography coupled with mass spectrometry (LC-MS) [80,81,82]. As with GC, the column contains the stationary phase, while a liquid mobile phase carrying the analyte flows through the column. The relative affinity of the analyte for the mobile and stationary phases allows for its separation from other matrix constituents. Finally, the mass spectrometry module is used to identify the analyte based on its molecular weight. 

An alternative approach to hyphenated techniques is coupling GC or LC separation to FTIR detection. This allows individual compounds to be separated in the gas or liquid phase, before collecting FTIR spectra from each eluting compound, providing detailed structural information on the functional group of the analyte(s). GC-FTIR has proved to be effective in identifying and quantifying separated compounds in foodstuffs [83,84]. More recently, the use of a new FTIR interface allowed the detection, identification and quantification of trace components at the nanogram level [85,86]. The use of the same interface coupled to liquid chromatography [87] opens the way to more applications for LC-amenable constituents. 

In cases where the compounds of interest are known and pure standards are available for comparative purposes, high-performance liquid chromatography (HPLC) with ultraviolet–visible detection may suffice [88]. This method works in the same way as LC-MS, but uses absorbance in the ultraviolet–visible region to detect the eluting compounds, rather than mass spectrometry. 

Colorimetric methods, such as the Folin–Ciocalteu assay, may also be used for the analysis of total phenolics, or for the quantification of anthocyanins using the pH differential method [89]. However, these methods are less specific compared to separation-based techniques, such as HPLC and GC-MS. 

More recently, there has been interest in using rapid, non-invasive, stand-alone analytical techniques, such as IR spectroscopy, for the prediction of bioactive compounds [90,91,92]. This emerging area of research is the focus of this review.

### 2.5. Previous Work and Aims

Although several previous reviews have focused on the use of IR spectroscopy for the estimation of specific groups of bioactive compounds, such as antioxidants [93,94] and phenolics [95], there are no contemporary reviews in the last decade on this technique for the quantification of bioactive compounds in food products. For instance, the review by McGoverin et al. [96] on this topic is over ten years old, with numerous IR-related papers published during the ensuing period. Similarly, the review by Pallone et al. [97] on the use of vibrational spectroscopy in food analysis included only seven studies quantifying constituents which could be classified as “bioactive” compounds. Hence, this paper aims to review the contemporary literature reporting the estimation or quantification of bioactive compounds in food matrices.

## 3. Methods

The Scopus database (https://www.scopus.com/; accessed on 11 October 2021) was used to search for articles between 2016–2020 containing the following terms in their title, abstract or keywords sections:

- Any of the following: near infrared OR mid infrared OR spectroscopy;
- AND food;
- AND bioactive OR phenolic OR antioxidant OR anthocyanin;
- AND quantification OR determination OR measurement.

In this way, articles pertaining to the quantification of bioactive constituents of functional foods using infrared spectroscopy were listed. 

Articles up to and including 31 December 2020 were considered, with the search limited to articles published in the 5 years prior (i.e., 1 January 2016 to 31 December 2020). The titles and abstracts of all articles were manually screened to find relevant articles for inclusion in this review. 

Inclusion criteria were as follows:

- Original studies published in the last 5 years (between 2016 and 2020);
- Quantified a compound or group of compounds with recognised health-benefiting effects, above that expected from basic nutritional needs;
- The matrix was a food or potential food product.

## 4. Scientific Effort (2016–2020)

The scientific effort over the past five years is summarised in Table 2 (for NIRS) and Table 3 (for MIRS). The information presented in the tables includes the type of matrix analysed, analyte(s) investigated, sample size of the calibration and validation sets, wavelength range used in the optimised model, and statistical method used for analysis of the spectra. All fruit and vegetable samples were analysed fresh and intact, unless otherwise stated in the table. The test set column shows whether the authors used a dependent test set for the model validation (i.e., samples from the same population as the calibration set) or independent test set (i.e., samples drawn from a different population to the calibration set, such as from a different year, season or geographic location). The cross-validation statistics (R^2^_CV_ and RMSECV) are reported in the corresponding columns for all studies. In cases where the study also included an independent test set, the R^2^_p_ and RMSEP for the test set are reported in the test set column. Finally, the notes column provides information about the sample population details and notable findings of the study. 

### 4.1. General Trends

#### 4.1.1. Publications by Year

Between 2016 and 2020, an average of 10 studies per year were published on the use of infrared spectroscopy for the measurement of bioactive compounds in food products. The number of studies published per year over this period remained relatively constant, with 13 papers in 2016, 12 in 2017, 10 in 2018 and 11 in 2020. However, in 2019, only 3 studies were found. 

#### 4.1.2. Matrix Type

Interrogation of the included studies by matrix type revealed that NIR spectroscopy was most commonly used for the analysis of bioactive compounds in fruit matrices, followed by aromatic plants, grains/pulses and beverages (Table 4). In contrast, MIR spectroscopy was most often reported for the analysis of beverages, likely due to the ease of presentation for this sample type.

#### 4.1.3. Optical Geometry

The majority of publications (58%) using NIR spectroscopy for the prediction of bioactive compounds used reflectance or diffuse reflectance geometry. A further 16% of studies used hyperspectral imaging in reflectance mode. Only 20% of studies used transmittance and 9% used transflectance, the majority of which were performed on beverage or oil samples. However, it should be cautioned that the vast majority of studies were not validated through independent test set validation and hence have not shown their robustness in “real-world” use; consequently, the optical geometry types used in the academic studies reported here may not reflect the optical geometry of instruments used commercially. 

All of the MIR spectroscopy studies except one [92] used an Attenuated Total Reflection (ATR) sampling platform, which requires samples to be placed in close contact with the ATR crystal. In general, studies comparing both NIRS and MIRS tended to show similar accuracy between these two techniques. The simple sample preparation for MIRS—particularly when using MIR-ATR—combined with its generally high accuracy would seem to make it suitable for a wide range of applications. 

#### 4.1.4. Sample Size and Test Sets

The number of calibration samples ranged from 10 to 387 (mean = 83 ± 72 samples), while the size of the validation set ranged from 5 to 182 (mean = 37 ± 32 samples). The majority of studies used a dependent test set (65%) or did not use any test set (24%), while only 9% of studies used an independent test set for validation of the developed model. 

Within the four NIRS studies utilising an independent test set, one used transmittance [112], while the others used reflectance [13,98] or diffuse reflectance geometry [109]. Cunha Júnior et al. [98] sourced their test set from the following season to the calibration set, while Ncama et al. [13] used test set samples from a geographically distinct farm (~400 km away) and Cozzolino et al. [109] used commercially sourced samples for validation purposes. The study by Tilahun et al. [112] could arguably be classified as using a semi-independent test set, as the authors used samples from a different harvest time point within the same season and from the same location. Interestingly, all four of these NIRS studies were performed on fruit rather than other food matrices. 

In most of these studies, the test set validation statistics were moderately poorer compared to the cross-validation statistics. For example, Cunha Júnior, et al. [98] found an R^2^_CV_ of 0.89–0.91 and RMSECV of 2.5–2.9 g/kg, compared to an R^2^_p_ of 0.74–0.88 and RMSEP of 5.1–6.8 g/kg. Similarly, the RMSEP for the prediction of lycopene content in tomato fruit was moderately higher at 1.79 mg/kg compared to the RMSECV of 1.56 mg/kg [112]. However, the performance of the test set from Cozzolino, et al. [109] was much worse, with an R^2^_p_ of 0.73 and RMSEP of 4733 mg/100 g (compared to an R^2^_CV_ of 0.93 and RMSECV of 1839 mg/100 g for cross-validation). 

Using MIRS for the analysis of chocolate samples, Hu, et al. [31] found that the test set statistics for the prediction of (+)-catechin, (+)-epicatechin and total phenolics in chocolate using MIRS were quite comparable to the cross-validation statistics. However, the RMSEP for prediction of total antioxidant capacity (TAC) in the same samples was 3–12 times higher than the RMSECV, suggesting that MIRS was not suitable for the accurate estimation of TAC in this matrix. These few examples illustrate the level of over-optimistic results which are likely to be purported when using no test set or a dependent test set for model validation.

#### 4.1.5. Chemometric Techniques

Nearly all of the publications used partial least squares (PLS) regression or some derivative of this regression technique for model development. Tschannerl et al. [118] used Support Vector Regression (SVR), a quantitative form of Support Vector Machines (SVM), for the prediction of total phenolic content in barley malt samples. However, only 10 samples were investigated in that study, with no independent test set used. Zhang et al. [106] also used SVR for the prediction of phenolic content in wine grape skins and seeds, demonstrating that for most analytes, the use of SVR gave better results than PLS or principal component regression (PCR). Xiao et al. [102] used a Least Squares Support Vector Machine (LS-SVM) algorithm for the prediction of total phenolics in white and red grapes, again with better results found compared to the standard PLS algorithm. Finally, Ding, et al. [113] compared the use of Radial Basis Function Neural Networks (RBF-NN) and PLS in dehydrated tomato samples, finding that RBF-NN performed better for the lycopene, total phenolic content and total antioxidant capacity measured by the DPPH and ABTS assays, while PLS performed better for the prediction of total antioxidant capacity via the FRAP method. Furthermore, only this one study used a deep learning or ANN algorithms (in this case, RBF-NN), although machine learning is a topic of increasing interest for other areas of IR spectroscopy [149,150].

### 4.2. Trends by Analyte Class

Another major aspect of interest to researchers in this field is the types of bioactive analyte(s) that have been measured using IR spectroscopy. Consequently, Table 5 presents a break-down of the studies included in this review by the compound class of the reported analytes. Additionally, the major classes are discussed in the following sections.

#### 4.2.1. Polyphenols

The greatest number of studies examined for the purpose of this current review were focused on predicting the total polyphenol content, or the content of specific polyphenol compounds present in the matrix (Table 5), with over half of all investigations focused on these analytes. There is an ongoing interest in biochemical characterisation and quantification of polyphenols across a wide range of food products, given that compounds from this class have been associated with a wide range of potential health-benefiting effects [151,152,153,154,155], particularly in improving cardiovascular health [156,157,158,159,160]. Consequently, the rapid prediction of total polyphenol content using infrared spectroscopy could have the potential to greatly benefit the effectiveness and robustness of the quality assurance of functional food products [40,96]. 

Ferrer-Gallego et al. [161] provided a recent review of the use of vibrational spectroscopy in the prediction of the phenolic composition of grapes and wines, although other food matrices were not considered in their review. The authors considered that this technique showed considerable promise for this purpose, although noted that future studies on grapes and wine should incorporate a wider range of environmental and genotypic variation. 

Some authors have reported difficulty in creating robust models for the prediction of total polyphenols using infrared spectroscopy. For example, Martín-Tornero et al. [162] found that NIRS and MIRS could only be used as a screening method for the total polyphenol content in grape leaves, due to the high prediction errors associated with the models created. These authors used a dependent test set (leaves collected from different locations within the same vineyards). In blackberry fruit, the best model for total phenolics reported by Toledo-Martín et al. [100] had a R^2^_CV_ of 0.69 and RMSECV of 169 mg/100 g. Again, the cross-validation samples used in this study were randomly selected from the same population as the calibration samples; consequently, the model performance on an independent population would be lower again. Similar results in terms of model accuracy were found by Rodríguez-Pulido et al. [110] in raspberries, Trapani et al. [127] in olive paste and Hernández-Hernández et al. [131] in cocoa bean, while quite poor cross-validation results were found by Nogales-Bueno et al. [133] for the prediction of total phenolic content (TPC) in coffee bean using NIR hyperspectral imaging. As the mean TPC of the samples was 3.6% *w*/*w*, the poor performance appears more attributable to the reproducibility of sample presentation or the wavelength selection, rather than the concentration of the analyte. 

In contrast, Tzanova et al. [101] and Jara-Palacios et al. [105] reported quite good findings for the prediction of total polyphenol content in grapes and grape pomace, respectively (R^2^_CV_ = 0.87–0.97; RMSECV = 9.6–21 mg/100 g), indicating that the instrument choice, geometry and data processing techniques may have an influence in addition to the matrix type. However, it is important to note that none of the aforementioned studies on the prediction of total phenolic content used an independent test set; therefore, the results should be taken with caution. 

There do not appear to have been any studies that focused on the IR quantitation of specific phenolic compounds or total phenolic content in model systems; hence, it is difficult to know what limit of detection and level of error to expect when using IR spectroscopy for this purpose. Although Abbas, et al. [9] used MIRS for the qualitative identification of 36 phenolic compounds (presented in powder form), they did not attempt the quantitation of these compounds in a model matrix.

#### 4.2.2. Anthocyanins

The second-most common analyte type that has been investigated using infrared spectroscopy was anthocyanins. Most of these studies (13 out of 17) looked at the total anthocyanin content, while only 4 studies attempted the prediction of specific anthocyanins. As a class of flavonoids, anthocyanins are less abundant than total polyphenols, so would be expected to be a more challenging target for infrared spectroscopy. Anthocyanins are brightly coloured and absorb light at around 520 nm; hence, it may be thought that they could be detected using the visible wavelengths of Vis–NIR instruments. However, surprisingly, all except one of the studies using NIRS for the measurement of anthocyanins did not include the visible light region in the optimised models, indicating that the infrared region actually contained most of the functional information pertaining to the anthocyanin content. Given the low concentration of anthocyanins, their prediction through NIRS is likely to rely upon secondary correlations with other matrix constituents.

Most studies using NIRS reported reasonably high accuracies for anthocyanin prediction in fresh sample matrices (R^2^_CV_ = 0.72–0.98; RMSECV = 9–13 mg/100 g), while MIRS performed similarly well for the estimation of anthocyanin content in soybean, grape juice and red wine. Additionally, Rodríguez-Pulido et al. [110] found that there was a reduced model linearity using NIRS in raspberry fruit (R^2^_CV_ = 0.63), although the RMSECV obtained was roughly comparable at 12 mg/100 g. 

Studies attempting the prediction of individual anthocyanins in red grapes [103] and wine [92,147] found that the concentrations of most of these compounds could be predicted with only slightly lower accuracy compared to the total anthocyanin content. Given the very low concentrations of many of these compounds, it is likely that the created models were indirectly measuring their concentration via their correlation with other, more abundant compounds which are more readily detected using infrared spectroscopy (possibly the predominant individual anthocyanin compounds present in the sample). Somewhat confusingly, many of the studies reported the anthocyanin content in units of mg/L of the sample extracts, rather than being correctly reported in mg/g or mg/100 g of the intact fruit from which the infrared spectra were obtained. Hence the results of these models should be interpreted with some degree of prudence. Future researchers in this area should be aware of and avoid this common pitfall.

#### 4.2.3. Carotenoids

In contrast to the trends observed for anthocyanins, studies investigating carotenoids using infrared spectroscopy mainly attempted the prediction of specific carotenoid compounds (β-carotene, lycopene) compared to those predicting the total carotenoid content. Additionally, all of the studies attempting carotenoid prediction were performed using NIRS. 

Several studies in intact fresh tomato fruit reported similar results for the prediction of lycopene (R^2^_CV_ = 0.85–0.86; RMSECV = 18–103 mg/100 g FW) and β-carotene (R^2^_CV_ = 0.71–0.77; RMSECV = 10–114 mg/100 g FW) [111,112]. Similar results were found for lycopene in dried tomato powder [113]. 

Toledo-Martín et al. [100] also found acceptable results for the total carotenoid content in blackberry (R^2^_CV_ = 0.76, RMSECV = 0.01 mg/100 g), with the carotenoid model outperforming that developed for total phenolic content in the same crop. Higher model accuracies (R^2^_CV_ > 0.9; RMSECV < 0.01 mg/100 g) were reported for β-carotene content in carrot [115] and marsh grapefruit [13], as well as for total carotenoids in honey [139].

#### 4.2.4. Ascorbic Acid

Studies using infrared spectroscopy (NIRS or MIRS) for the estimation of ascorbic acid content were performed in Kakadu plum powder [109], carrot [115], frozen guava pulp [107], cashew apple and guava nectar [135], and soft drinks [138]. Most models showed reasonable accuracy (R^2^_CV_ = 0.7–0.98; RMSECV = 4–7 mg/100 g). Due to the exceptionally high ascorbic acid content in Kakadu plum (mean content of 14,323 mg/100 g), the RMSECV values of Cozzolino et al. [109] were much higher at 1811–1839 mg/100 g. The model linearity was quite high (R^2^_CV_ = 0.91–0.93), with an RPD of 4.0–4.1, although the independent test set validation (comprising commercially purchases samples of Kakadu plum powder) gave a high RMSEP. All of the other aforementioned studies did not validate their models using independent test sets, but only used dependent test sets (comprising randomly selected samples from the full dataset). 

The study by Cozzolino et al. [109] was also the only study to compare the performance of NIRS and MIRS for predicting ascorbic acid content, finding a slightly improved accuracy of NIRS compared to MIRS in dried Kakadu plum powder.

#### 4.2.5. Other Analytes

Other bioactive compounds assessed using infrared spectroscopy included chlorophylls, fatty acid esters, squalene and tocopherols (compounds related to vitamin E) in olive oil, piperine in black pepper, caffeine in black tea, and theobromine in cocoa bean. In general, good results were generally found for caffeine, and most tocopherols and fatty acids, while moderately accurate results were found for theobromine, squalene, chlorophylls and piperine (R_2CV_ = 0.7–0.8). However, it should be noted that most of these analytes were only investigated in a single study. Nevertheless, these results support the use of infrared spectroscopy as an adaptable tool for the rapid estimation of a substantially wide range of bioactive compounds in food-based matrices.

### 4.3. Future Directions

As found throughout this review, IR spectroscopy shows considerable potential for the quantification and relative prediction of the levels of bioactive components in food matrices. Although the research has mostly been presented as proof-of-concept work and/or conducted under controlled laboratory conditions, interest and applications in this field are likely to continue to grow. A brief discussion on several particular aspects worth noting is provided here. 

Hyperspectral imaging is a rapidly growing area of research in the food science sector, particularly for the determination of food quality and safety [163,164,165,166], but also for authentication purposes [167,168]. This technique can collect near-infrared spectra from each pixel in a photograph (creating a ‘hypercube’ dataset), allowing for analysis of the spatial variation of the analyte, in addition to its mean concentration in the sample. Consequently, hyperspectral imaging could potentially be used for the quantification of bioactive compounds [169]. Indeed, several of the studies reviewed here applied hyperspectral imaging for the estimation of anthocyanins and phenolic acids in grapes and grape byproducts [103,104,105,106], and for the estimation of phenolics in barley malt [118] and coffee beans [133]. However, there have been limited applications of hyperspectral imaging systems in industrial applications to date, due to its associated challenges such as obtaining reproducible sample presentation, minimising the effects of ambient light, and the complexity of data analysis [166]. Furthermore, hyperspectral imaging can only be used with a reflectance geometry. Additionally, the cost of these instruments remains quite high; thus, they are only used for applications which have a need for spatial information. 

The use of IR spectroscopy as a real-time, online (or “inline”) process analytical technology is another principal area of interest. NIRS is already commonly used in manufacturing environments and processing plants for the online analysis of a range of food products, principally for the determination of proximate quality parameters, such as moisture/dry matter content, soluble solids and protein [170,171]. This real-time information can then be fed back into the manufacturing system, allowing various processing parameters to be adjusted accordingly in view of maintaining the optimal quality of the product. With emerging interest in bioactive compounds in functional food products, online NIRS could potentially be extended to the quality assurance of the presence of these compounds in addition to existing analytes already being monitored. 

Finally, it is worth noting the importance of confirming the accuracy and reproducibility of infrared spectroscopy techniques using sufficiently large sample sizes and test sets which are independent to the calibration sets. Given that only a small fraction of the studies reviewed here used a fully independent test set for model validation purposes, it is likely that the reported accuracy is in many instances quite over-optimistic and not representative of the true accuracy which could be expected if applying the model for routine quality assurance purposes.

## 5. Conclusions

The technique of IR spectroscopy has enjoyed considerable success in the food analysis industry over the past few decades. In recent years, an increasing number of studies are exploring the use of this technology for the analysis of bioactive compounds in food products, such as polyphenols, anthocyanins or carotenoids. While much reported work is still in the proof-of-concept or method development stage, IR spectroscopy appears to show promise for the relative assessment—if not absolute quantification—of these bioactive analytes. In particular, the ease of sample preparation and reasonable accuracy of MIR-ATR (comparable to NIRS in many studies) would appear to make this technology suitable for a wide range of applications in the food industry. 

## Figures and Tables

**Table 2:** Studies reporting the use of near-infrared spectroscopy for the quantification of bioactive compounds in food products (2016–2020).

Food Matrix	Analyte(s)	Sample Size (cal/val)	Wavelength Range (nm)	Optical Geometry	Statistical Method	Test Set	Cross-Validation	R^2^_CV_	RMSECV	Notes	Reference
Fruit											
Açaí and juçara	Total anthocyanin content	Variable (*n* = 374 total)	1606–1793	Reflectance	PLS	Independent populations R^2^_p_ = 0.74–0.88; RMSEP = 5.09–6.76 g/kg	LOO	0.89–0.91	2.50–2.91 (g/kg)	Fruit from two seasons and four localities	[98]
Bilberry (dried powder)	Anthocyanins	38/27	1064–1640, 1833–2354	Reflectance	PLS	Dependent test set (randomly selected samples)	LOO	0.995	0.28 (% *w*/*w*)	NIR analysis could identify counterfeit bilberry samples	[99]
Blackberry	Total phenolics Total carotenoids	90/30	400–2500	Reflectance	PLS	None	n/s	0.69 0.76	1.69 0.95 (mg/g)		[100]
Grapes (red)	Trans-resveratrol Quercetin Total phenols	15	900–1700	Reflectance	PLS	None	LOO	0.988 0.955 0.974	0.424 mg/kg 0.008 mg/kg 12.15 mmol/kg	Three locations; two seasons	[101]
Grapes (red and white)	Total phenolics	203/67	400–1100 900–2500	Reflectance	PLS, SVM	Dependent test population	n/s	0.872–0.914 0.697–0.726	0.15–0.22 0.28–0.31 (mg/g)	Two cultivars from one season and location; SVM gave better results than PLS	[102]
Grapes (red)	Total anthocyanins Total 3-O-glucoside anthocyanins Total 3-O-(6-acetyl)glucoside anthocyanins Total 3-O-(6-p-coumaroyl)glucoside anthocyanins Malvidin 3-O-glucoside Malvidin 3-O-(6-acetyl)glucoside Malvidin 3-O-(6-p-coumaroyl)glucoside Petunidin 3-O-glucoside Petunidin 3-O-(6-acetyl)glucoside Petunidin 3-O-(6-p-coumaroyl)glucoside Delphinidin 3-O-glucoside Delphinidin 3-O-(6-acetyl)glucoside Delphinidin 3-O-(6-p-coumaroyl) glucoside Peonidin 3-O-glucoside Peonidin 3-O-(6-acetyl)glucoside Peonidin 3-O-(6-p-coumaroyl)glucoside Cyanidin 3-O-glucoside Cyanidin 3-O-(6-acetyl)glucoside Cyanidin 3-O-(6-p-coumaroyl)glucoside	60/20	380–1028	Reflectance (hyperspectral imaging)	MPLS	Dependent test set (stratified samples)	Six-fold cross-validation	0.91 0.92 0.90 0.83 0.87 0.90 0.80 0.93 0.57 0.91 0.91 0.92 0.88 0.80 0.75 0.88 0.77 0.86	189.05 155.94 4.12 23.09 73.93 4.13 13.32 29.44 0.98 2.04 48.41 3.28 30.35 0.31 3.81 16.43 0.16 1.78 (mg/L)	Eight different cultivars from two sites	[103]
Grapes (red)	Nonacylated anthocyanins Total anthocyanins	47/-	950–1650	Reflectance (hyperspectral imaging of single grapes)	PLS	Dependent test population	LOO	0.72 0.72	0.78 0.70 (mg/grape)	Fruit from two dates and two vineyards within one season	[104]
Grape pomace (marc)	Catechin Epicatechin Proanthocyanidin B1 Proanthocyanidin B2 Proanthocyanidin B3 Proanthocyanidin B4 Proanthocyanidin trimer 1 Proanthocyanidin trimer 2 Proanthocyanidin tetramer 1 Proanthocyanidin tetramer 2 Proanthcyanidin B2-3-O-gallate Galloyl proanthocyanidin Total flavanols Gallic acid Protocatechuic acid Caffeic acid Caftaric acid Cis-coutaric acid Trans-coutaric acid Total phenolic acids Quercetin 3-O-rutinoside Quercetin 3-O-glucuronide Quercetin 3-O-glucoside Quercetin pentoside Kaempferol 3-O-galactoside Kaempferol 3-O-glucuronide Kaempferol 3-O-glucoside Quercetin Kaempferol Total flavonols	12/-	950–1650	Reflectance (hyperspectral imaging)	PLS	None	LOO	0.80 0.96 0.65 0.75 0.50 0.63 0.65 0.86 0.65 0.53 0.89 0.58 0.78 0.75 0.82 0.92 0.91 0.83 0.95 0.87 0.63 0.81 0.64 0.15 0.98 0.93 0.98 0.72 0.97 0.70	14.00 4.72 20.53 1.86 3.43 3.01 3.12 7.68 11.62 2.66 6.29 7.27 66.63 5.58 2.70 0.36 2.56 0.15 0.19 9.61 1.82 4.36 5.95 0.04 0.11 0.07 0.41 0.19 0.02 14.27 (mg/100 g)	Fruit from one variety, season and location	[105]
Grape skins (red)	Total iron-reactive phenolics Anthocyanins Tannins	40/20	977–1625	Reflectance (hyperspectral imaging)	PCR, PLS, SVR	Dependent test set	Segment validation	0.907 0.879 0.896	0.178 0.144 0.107 (mg/L)	Five cultivars from four dates in one growing season	[106]
Grape seeds (red)	Total iron-reactive phenolics Tannins	40/20	977–1625	Reflectance (hyperspectral imaging)	PCR, PLS, SVR	Dependent test set	Segment validation	0.879 0.924	0.240 0.519 (mg/L)	Five cultivars from four dates in one growing season	[106]
Guava (frozen pulp)	Ascorbic acid	50	1000–1892, 2007–2227	Transflectance	PLS	Dependent test set (randomly selected samples)	LOO	0.85	6.14 mg/100 g (test set)	Samples from two Brazilian marketplaces	[107]
Jujube	Gallic acid Caffeic acid L-epicatechin Phloridzin Cianidanol	52/26	900–1700	Transmittance	Si-ACO-PLS	Dependent test population	n/s	0.879 0.887 0.906 0.858 0.836	3.06 6.04 16.30 0.84 16.01 (µg/g)	Samples from five regions	[108]
Kakadu plum (powder)	Ascorbic acid	80/5	866–2532	Diffuse reflectance	PLS	Independent (commercially sourced samples) R^2^_p_ = 0.73; RMSEP = 4733 mg/100 g	n/s	0.93	1839 mg/100 g		[109]
Marsh grapefruit	β-Carotene Total carotenoids	240	850–2500 400–850	Reflectance	PLS	Independent (samples from a separate orchard)	Test set validation	0.99 0.92 (test set)	0.00 2.69 (µg/g) (test set)	Fruit from one season and two locations	[13]
Raspberries	Total phenols Total anthocyanins TAC (FRAP)	168	950–1650	Reflectance (hyperspectral imaging)	PLS	None	n/s	0.70 0.63 0.61	127 12 39 (mg/100 g)		[110]
Tomato	Total phenolics Lycopene Total flavonoid β-Carotene	50	285–1200	Reflectance	PLS	None	LOO?	0.834 0.864 0.790 0.708	1.80 1.03 1.82 1.14 (µg/g)		[111]
Tomato	Lycopene β-Carotene	180/60	500–1100	Transmittance	PLS	Semi-independent (separate harvest in same season) R^2^_p_ = 0.85, 0.77; RMSEP = 1.79, 1.00 mg/kg	LOO	0.89 0.88	1.56 0.63 (mg/kg)		[112]
Tomato (dehydrated and ground)	Lycopene Phenols TAC (DPPH) TAC (FRAP) TAC (ABTS)	61/31	800–2500	Reflectance	PLS, RBF-NN	Dependent test set	n/s	0.882 0.910 0.882 0.876 0.937	1.61 80 0.70 0.97 0.86 (mg/100 g)	RBF-NN generally performed better than PLS regression	[113]
Wax jambu	Total phenolics Total anthocyanins	50/35	1000–2400	Diffuse reflectance	PLS	Dependent test set (Kennard-Stone selection)	n/s	0.94 0.98	22.18 9.0 (mg/100 g)		[114]
Vegetables											
Carrot	Ascorbic acid β-Carotene	24/6?	420–1100	Reflectance	PLS	Dependent test set	Four-fold cross-validation	0.98 0.98	0.04 µg/g 0.10 µg/100 g	Roots sampled over an 8-week storage period	[115]
Red cabbage (EtOH extract)	Total anthocyanins Monomeric anthocyanins Total polyphenols	1 (with 33 serial dilutions)	1000–2500	Transmittance	PLS	9 dilutions prepared from new cabbage extract	Segment validation	0.98 0.98 0.96	16.4 mg/L 20.2 mg/L 42.7 mg/L		[116]
Potato	Total phenolics Antioxidant capacity (DPPH)	160/68	1100–2300	Reflectance	PLS	Dependent test set	Venetian blind cross-validation	0.84 0.67	1.20 1.21 (mg/g)	Included white, red, yellow and purple-fleshed cultivars	[117]
Grains/pulses											
Barley malt	Total phenolics	10	1000–2500	Reflectance (hyperspectral imaging)	SVM, SVR	Dependent test set (5% of total pixels)	n/s	0.85	1 ppm		[118]
Buckwheat, oat, millet	Total phenolics	77	1596–2396 1128–2162 740–1070	Reflectance	PLS	Test set used but no information provided on its origins or size	LOO (for most models)	0.921 0.951 0.823	1.46 1.11 1.98 (mg/g)	Compared three handheld instruments (microPhazir RX, MicroNIR 2200, SCiO)	[119]
Common bean (flour)	Total phenols Ortho-diphenols Flavonoids Gallic acid Catechin Myricetin-3-glucoside Quercetin-3-6″-manolyl-glucoside Kaempferol-3-glucoside Kaempferol-3-6″-manolyl-glucoside Kaempferol	42/-	1000–2500	Reflectance	PLS	Spectra randomly selected from dataset (1/3 of total spectra)	LOO	0.91 0.85 0.90 0.96 0.48 0.97 0.90 0.85 0.93 0.87	RPDs: 5.20 4.84 5.18 10.25 2.38 10.25 7.52 6.27 9.32 6.51	21 varieties; two seasons	[120]
Mungbean	Catechin Chlorogenic acid Caffeic acid p-coumaric acid t-ferulic acid Vitexin Isovitexin Myricetin Quercetin Kaempferol	42/18	1600–2500	Reflectance (from whole grains)	PLS	Dependent test set	Segment validation	0.996 0.998 0.992 0.989 0.998 0.997 0.997 0.994 0.989 0.998	0.603 0.590 1.78 1.8 0.519 0.238 0.23 1.82 1.67 0.5 (%)		[121]
Quinoa (whole seed)	Total free phenolics Total betalains TAC (DPPH)	38/-	400–2500	Reflectance	PLS	None	Segment validation	n/s n/s 0.73	n/s n/s 8.6 (mmol/kg)	For TAC in ground seed; R^2^_CV_ = 0.66; RMSECV = 9.6 mmol/kg	[122]
Soybean	Total anthocyanins Cyanidin-3-glucoside Delphinidin-3-glucoside	70	1000–2500	Reflectance	PLS	Subset of spectra of samples included in calibration set	n/s	0.88 0.90 0.88	0.13 0.12 0.03 (mg/g)		[123]
Oils											
Olive oil	Squalene	118/59	1100–2300	Transmittance	PLS	Dependent test set	LOO?	0.83	2.31 (g/kg) (pred)	Poorer results obtained using Vis–NIRS data	[124]
Olive oil	Total tocopherols α-Tocopherol β-Tocopherol γ-Tocopherol	197/91 189/93 197/102 195/101	350–2500	Transmittance, transflectance	PLS	Dependent test set	LOO?	0.89 0.92 0.54 0.85	43.83 33.90 0.59 4.54 (mg/kg) (SEC)	Vis–NIRS gave slightly better results than NIRS in most cases	[125]
Olive oil	Tyrosol Tyrosol secoiridoids Hydroxytyrosol Hydroxytyrosol secoiridoids Total phenolics	75/18	800–2500	Transmittance	PLS	None	LOO	0.55 0.84 0.55 0.82 0.82	5.27 41.5 4.84 43.1 76.7 (mg/kg)		[126]
Olives (as paste)	Total phenolics Oleuropein	291/53 147/53	1400–2400	Reflectance	PLS	Dependent test set	LOO?	0.71 0.73 (cal)	0.08 6.6 (mg/kg)	Samples obtained across seven seasons	[127]
Aromatic plants											
Black pepper (whole)	Piperine	132/-	950–1650	Reflectance	PLS	None	Segment validation	0.726	0.289g/100 g	For ground samples; R^2^_CV_ = 0.850, RMSECV = 0.231g/100 g	[128]
Black tea	Caffeine Epigallocatechin gallate	37/19	950–1650	Reflectance	PLS, MLR	Dependent test set	LOO?	0.933 0.782	3.65 3.32 (mg/g)		[129]
Black tea	Cianidanol Ferulic acid Gallic acid Rutin Phloridzin L-epicatechin	84/56 (20 replicate samples at seven time points)	899–1724	Transmittance	CARS-PLS	Dependent test set	n/s	0.956 0.928 0.911 0.825 0.881 0.969	9.66 0.21 4.22 0.77 6.85 20.1 (mg/100 g)	20 tea samples collected at seven time points during fermentation process	[130]
Cocoa bean	Total phenols Catechin Epicatechin Epigallocatechin Theobromine	74/- 76/- 75/- 72/- 75/-	400–2498	Reflectance	PLS	None	LOO?	0.71 0.62 0.04 0.02 0.77	6.09 0.65 5.24 0.09 4.55 (mg/g)		[131]
Cocoa bean	Total polyphenols	72	800–2778	Diffuse reflectance	PLS	None	LOO	0.84	0.93 (mg/g)	Sample variation induced by different periods of storage and fermentation	[132]
Cocoa bean husk	Total phenols Catechin Epicatechin Epigallocatechin Theobromine	77/- 80/- 79/- 78/- 78/-	400–2498	Reflectance	PLS	None	LOO?	0.81 0.74 0.06 0.20 0.83	4.75 0.55 5.31 0.10 3.72 (mg/g)		[131]
Coffee bean	Chlorogenic acid Total phenolics	101/36	950–1650	Reflectance (hyperspectral imaging)	MPLS	Dependent test set	n/s	0.81 0.58 (cal)	0.91 4.63 (mg/g) SEP = 15.6 and 17.6%		[133]
Ginger	Zingerone 6-Gingerol 8-Gingerol 10-Gingerol 6-Shogaol	58/22	1389–2500	Reflectance	PLS	Dependent test set	LOO	0.981 0.986 0.988 0.997 0.998 (cal)	0.076 0.072 0.078 0.077 0.084 (mg/g)		[134]
Beverages											
Cashew apple nectar	Ascorbic acid	49/16	1000–1903, 1971–2227	Transflectance	PLS	Dependent test set (randomly selected samples)	n/s	0.84 (cal)	4.8 mg/100 g (test set)	Samples from two Brazilian marketplaces	[135]
Coffee aqueous solution	Chlorogenic acid	86	401–1871	Transmittance (1 mm path length)	PLS	None	LOO	0.556	0.76 mg/mL	Key predictor wavelength was around 1450 nm (C-H vibration; second overtone)	[136]
Grape juice	Total phenolics Anthocyanins	49/16	1000–2500	Transflectance	PLS	Dependent test set (randomly selected samples)	Optimising no. of latent variables	0.96 0.84 (cal)	37 4.44 (mg/100 mL) (test set)	Slightly worse results for phenolic content compared to MIR	[137]
Guava nectar	Ascorbic acid	41/13	1000–1899, 1983–2227	Transflectance	PLS	Dependent test set (randomly selected samples)	n/s (LOO?)	0.86 (cal)	7.44 mg/100 g (test set)	Samples from two Brazilian marketplaces	[135]
Soft drink (grape and passionfruit)	Ascorbic acid	~47/20	1000–2500	Reflectance	PLS	Test set of 5 samples created by diluting one sample to specific concentrations	LOO	0.70 0.76	0.67 0.56 mg/g		[138]
Wine (red)	Trans-resveratrol Quercetin Total phenols	20	900–1700	Transmittance	PLS	None	LOO	0.994 0.990 0.996	0.113 mg/L 0.073 mg/L 0.144 mM	Three locations; two seasons	[101]
Wine (red)	Gallic acid Catechin B1 (flavonol dimer) Polymeric phenols Caftaric acid Caffeic acid Coutaric acid p-coumaric acid Quercetin-3-glucoside Quercetin Kaempherol Delphinidin-3-glucoside Cyanidin-3-glucoside Petunidin-3-glucoside Peonidin-3-glucoside Malvidin-3-glucoside Delphinidin-3-acetylglucoside Cyanidin-3-acetylglucoside Petunidin-3-acetylglucoside Peonidin-3-acetylglucoside Malvidin-3-acetylglucoside Delphinidin-3-cumarylglucoside Petunidin-3-cumarylglucoside Peonidin-3-cumarylglucoside Malvidin-3-cumarylglucoside Polymeric pigments MCP tannins Anthocyanins	~387/182	800–2500	Transmittance	PLS using PRESS	Dependent test set	Segment validation	0.86 0.83 0.76 0.88 0.86 0.87 0.84 0.87 0.88 0.84 0.85 0.92 0.86 0.9 0.85 0.87 0.88 0.91 0.92 0.91 0.85 0.86 0.85 0.86 0.84 0.86 0.92 0.87	3.01 5.85 4.94 135 8.8 0.82 2.63 0.61 10.3 1.65 0.15 2.32 0.05 2.16 1.73 16.5 0.65 0.34 0.89 0.65 7.15 0.19 0.57 0.84 4.27 5.71 204 53.1 (mg/L)	Wines comprised four cultivars from 13 vinifications over two seasons; more accurate at predicting phenolic content than ATR-MIR or transmission FT-IR.	[92]
Other foods											
Honey	Phenolics Flavonoids Carotenoids Antioxidants (FRAP)	105/45	1000–2500	Reflectance	PLS	Dependent test set (randomly selected samples)	Segment validation	0.884 0.903 0.922 0.922	14.5 1.01 0.035 0.43 (mg/100 g)	Six different floral varieties of honey	[139]
Propolis	Flavones and flavonols Flavanones and dihydroflavonols Antioxidant capacity (ABTS)	70/29	1100–2000	Reflectance (fibre-optic) on ground sample	MPLS	Dependent test set (randomly selected samples)	Segment validation	0.63 0.68 0.87 (cal)	29.4 9.5 112 (mg/g)	Samples sourced from Chile and Spain	[140]

Abbreviations: RBF-NN = radial basis function neutral network; LOO = leave-one-out cross-validation; n/s = not specified; PLS = partial least squares; SVM = support vector machine; TAC = total antioxidant capacity.

**Table 3:** Studies reporting the use of mid-infrared spectroscopy for the quantification of bioactive compounds in food products (2016–2020).

Food Matrix	Analyte(s)	Sample Size (cal/val)	Wavelength Range (nm)	Optical Geometry/Presentation	Statistical Method	Test Set	Cross-Validation	R^2^_CV_	RMSECV	Notes	Reference
Fruit											
Kakadu plum (powder)	Ascorbic acid	80/5	4000–400	ATR	PLS	Independent (commercially sourced samples) R^2^_p_ = 0.65; RMSEP = 2367 mg/100 g	n/s	0.91	1811 mg/100 g		[109]
Vegetables											
Red cabbage (EtOH extract)	Total anthocyanins Monomeric anthocyanins Total polyphenols	1 (with 33 serial dilutions)	4000–650	ATR	PLS	9 dilutions prepared from new cabbage extract	Segment validation	0.98 0.98 0.96	18.1 mg/L 21.3 mg/L 44.4 mg/L		[116]
Grains/pulses											
Buckwheat (leaves and flowers)	Rutin Quercetin Quercitrin Sum of flavonoids	Not stated (total = 108)	4000–500	ATR (whole and ground dried samples)	PLS	Dependent test set	LOO	0.99 0.99 0.95 0.98	3.63 0.06 2.48 4.80 (mg/g)	Used seven different species of buckwheat	[141]
Common bean (flour)	Total phenols Ortho-diphenols Flavonoids Gallic acid Catechin Quercetin-3-glucoside Quercetin-3-6″-manolyl-glucoside Kaempferol-3-glucoside Myricetin Kaempferol-3-6″-manolyl-glucoside Kaempferol	42/-	4000–400	ATR (flour)	PLS	Spectra randomly selected from dataset (1/3 of total spectra)	LOO	0.86 0.31 0.86 0.94 0.89 0.43 0.73 0.38 0.35 0.39 0.84	RPDs: 4.36 1.54 4.30 10.12 9.47 1.91 4.25 1.23 1.81 1.79 7.03		[120]
Soybean	Total anthocyanins Cyanidin-3-glucoside Delphinidin-3-glucoside	70/-	4000–650	ATR (whole seeds)	PLS	Spectra of samples included in calibration set	n/s	0.86 0.88 0.87	0.15 0.13 0.03 (mg/g)	70 different varieties	[123]
Oils											
Olive oil	Fatty acid methyl esters Fatty acid ethyl esters Fatty acid alkyl esters Diacylglycerols: C34 1,2 C34 1,3 C36 1,2 C36 1,3 Pheophytin a Chlorophyll a Pheophytin b Total xanthophyll Lutein Chlorophyll b	59/30	4000–650	ATR	PLS	Dependent test set	LOO?	0.87 0.85 0.87 0.62 0.83 0.79 0.77 0.72 0.75 0.71 0.61 0.75 0.72	41.63 27.43 60.10 (mg/kg) 1.07 1.26 4.29 4.02 (mg/kg) 2.42 0.32 0.10 0.41 0.71 0.21 (mg/kg)	Samples from two seasons; quite poor test set validation results for colour pigments; improved results from fusion of UV–Vis and IR spectra	[142]
Olive oil	Tyrosol Tyrosol secoiridoids Hydroxytyrosol Hydroxytyrosol secoiridoids Total phenolics	75/18	4000–400	ATR	PLS	None	LOO	0.32 0.30 0.17 0.19 0.44	4.98 105.7 9.96 106.1 162.1 (mg/kg)		[126]
Olive oil	Total phenolics	70/30	4000–600	ATR	PLS	Dependent test set	n/s	0.998	0.072 g/L		[143]
Beverages											
Cachaça	Total phenolics	32/16	4000–650	ATR (liquid sample)	PLS	Ranked subset of samples (60% cal; 20% val; 20% test set)	n/s	0.820	248 mg/L Much poorer results than fluorescence spectroscopy	For test set validation; R^2^_p_ = 0.690 and RMSEP = 318 mg/L	[144]
Grape juice	Total phenolics Anthocyanins	49/16	4000–400	ATR	PLS	Dependent test set (randomly selected samples)	Optimising no. of latent variables	0.90 0.81 (cal)	21 4.22 mg/100 mL (test set)	Performed better than NIR for phenolic content	[137]
Shiraz wine	Total anthocyanins Total phenolics	70/30	1700–950	ATR (liquid sample)	PLS	Dependent test set	LOO	0.61 0.60	32 mg/L 5.7 au	Wines from 24 different Australian locations	[145]
Wine (red)	Gallic acid Catechin B1 Polymeric phenols Caftaric acid Caffeic acid Coutaric acid p-Coumaric acid Quercetin-3-glucoside Quercetin Kaempherol Delphinidin-3-glucoside Cyanidin-3-glucoside Petunidin-3-glucoside Peonidin-3-glucoside Malvidin-3-glucoside Delphinidin-3-acetylglucoside Cyanidin-3-acetylglucoside Petunidin-3-acetylglucoside Peonidin-3-acetylglucoside Malvidin-3-acetylglucoside Delphinidin-3-cumarylglucoside Petunidin-3-cumarylglucoside Peonidin-3-cumarylglucoside Malvidin-3-cumarylglucoside Polymeric pigments MCP tannins Anthocyanins	~387/182	4000–600	ATR (liquid sample)	PLS using PRESS	Dependent test set	Segment validation	0.83 0.78 0.8 0.85 0.85 0.86 0.85 0.81 0.85 0.69 0.82 0.88 0.76 0.86 0.84 0.85 0.86 0.85 0.88 0.89 0.89 0.85 0.85 0.85 0.85 0.85 0.89 0.86	3.42 7.26 4.99 128 9.76 1.07 3.14 0.63 13 2.56 0.34 2.96 0.06 2.57 1.47 20.7 1.44 0.48 1.19 1.03 6.49 0.41 0.79 0.91 3.98 5.5 261 47.2 (mg/L)	Wines comprised four cultivars from 13 vinifications over two seasons; slightly less accurate at phenolic content compared to FT-NIR	[92]
Wine (red)	Gallic acid Catechin B1 Polymeric phenols Caftaric acid Caffeic acid Coutaric acid p-coumaric acid Quercetin-3-glucoside Quercetin Kaempherol Delphinidin-3-glucoside Cyanidin-3-glucoside Petunidin-3-glucoside Peonidin-3-glucoside Malvidin-3-glucoside Delphinidin-3-acetylglucoside Cyanidin-3-acetylglucoside Petunidin-3-acetylglucoside Peonidin-3-acetylglucoside Malvidin-3-acetylglucoside Delphinidin-3-cumarylglucoside Petunidin-3-cumarylglucoside Peonidin-3-cumarylglucoside Malvidin-3-cumarylglucoside Polymeric pigments MCP tannins Anthocyanins	~387/182	4000–600	Transmission	PLS using PRESS	Dependent test set	Segment validation	0.85 0.85 0.84 0.91 0.87 0.86 0.84 0.83 0.82 0.84 0.87 0.84 0.82 0.88 0.85 0.86 0.84 0.86 0.88 0.9 0.84 0.88 0.85 0.87 0.85 0.82 0.92 0.89	4.57 5.39 3.91 132 9.87 1.02 2.8 0.625 13 1.59 0.328 4.15 0.0645 4.1 2.1 24.2 1.28 0.513 1.24 1.12 8.85 0.463 0.831 1.01 4.7 7.49 224 56.5 (mg/L)	Wines comprised four cultivars from 13 vinifications over two seasons; slightly less accurate at phenolic content compared to FT-NIR	[92]
Wine (red, rose and white)	Total phenolics Total anthocyanins	35/-	4000–650	ATR	PLS	Cross-validation only	LOO	0.91 0.86	269.2 mg/L 1.79 mg/L	Seven wine samples (red, rose, white) each at five different time points	[146]
Wine (red and white)	Total polyphenols Malvidin-3-O-glucoside Peonidin-3-O-glucoside Petunidin-3-O-glucoside Delphinidin-3-O-glucoside Delphinidin-3-O-(6-acetyl)-glucoside Petunidin-3-O-(6-acetyl)-glucoside Peonidin-3-O-(6-acetyl)-glucoside Malvidin-3-O-(6-acetyl)-glucoside Delphinidin-3-O-(6-p-coumaroyl)-glucoside Malvidin-3-O-(6-p-coumaroyl)-glucoside o-coumaric acid	51/21	4000–650	ATR	PLS	Dependent test set	LOO	0.75 0.53 0.56 0.67 0.71 0.27 0.29 0.31 0.41 0.45 0.69 0.63	249.1 6.87 0.38 1.1 0.73 0.15 0.24 0.22 2.59 0.12 0.65 0.33 (mg/L)	Samples from various locations across two seasons	[147]
Other foods											
Chocolate	(+)-Catechin (+)-Epicatechin Total phenolics TAC (DPPH) TAC (ORAC)	18/7	4000–550	ATR	PLS	Semi-independent (7 randomly selected commercial chocolate brands) R^2^_p_ = 0.86, 0.72, 0.88, 0.89, 0.90; RMSEP = 0.10, 0.57, 5.08, 13.07, 37.92 mg/g	Nine-fold cross-validation	0.94 0.87 0.93 0.92 0.89	0.09 0.58 4.21 1.05 11.38 (mg/g)	18 different types of chocolate containing 35–100% cacao	[31]
Honey	Catechin Syringic acid Vanillic acid Chlorogenic acid TAC (DPPH)	64/36	3000–2800, 1800–700	ATR	PLS	Dependent test set (ranked subset of samples)	LOO	0.999 0.992 0.946 0.994 0.955	0.40 1.08 0.45 0.43 (µg/g) 1.63 (mg/100 g)	Models based on Raman spectra were slightly better than FTIR	[148]

Abbreviations: LOO = leave-one-out cross-validation; n/s = not specified; SFA = saturated fatty acids; MUFA = monounsaturated fatty acids; PUFA = polyunsaturated fatty acids; TAC = total antioxidant capacity.

**Table 4:** Number of studies included in this review, broken down by matrix type. If the same study used both NIR and MIR spectroscopy, it was counted separately in each column.

Matrix Type	Number of Published Studies	
	NIR	MIR
Fruit	18	1
Vegetables	3	1
Grains/pulses	6	3
Oils	4	3
Aromatic plants	7	0
Beverages	6	7
Others	2	2
Total	46	18

**Table 5:** Number of studies included in this review, broken down by analyte class. Note that if the same study investigated multiple matrices or investigated more than one analyte class in the same matrix, it was counted separately.

Analyte Class	Number of Published Studies
Total polyphenol content ^	34
Specific polyphenols ^	21
Total anthocyanin content	13
Specific anthocyanins	4
Total carotenoid content	2
Specific carotenoids (β-carotene, lycopene)	6
Ascorbic acid (vitamin C)	6
Alkaloids (theobromine, caffeine, piperine)	4
Fatty acid esters and other bioactive hydrocarbons	2
Chlorophylls	1
Tocopherols	1
Total	94

^ Polyphenols includes phenolic acids and flavonoid derivatives.

## Data Availability

The full dataset is available upon request from the corresponding author.

## References

[1] Almeida M., Torrance K., Datta A. (2006). Measurement of optical properties of foods in near-and mid-infrared radiation. Int. J. Food Prop..

[2] Burks C.S., Dowell F.E., Xie F. (2000). Measuring fig quality using near-infrared spectroscopy. J. Stored Prod. Res..

[3] Bureau S., Cozzolino D., Clark C.J. (2019). Contributions of Fourier-transform mid infrared (FT-MIR) spectroscopy to the study of fruit and vegetables: A review. Postharvest Biol. Technol..

[4] Walsh K.B., Blasco J., Zude-Sasse M., Sun X. (2020). Visible-NIR ‘point’ spectroscopy in postharvest fruit and vegetable assessment: The science behind three decades of commercial use. Postharvest Biol. Technol..

[5] Johnson J.B., Naiker M. (2019). Seeing red: A review of the use of near-infrared spectroscopy (NIRS) in entomology. Appl. Spectrosc. Rev..

[6] Velasco L., Schierholt A., Becker H.C. (1998). Performance of near-infrared reflectance spectroscopy (NIRS) in routine analysis of C18 unsaturated fatty acids in intact rapeseed. Lipid Fett.

[7] Bokobza L. (1998). Near Infrared Spectroscopy. J. Near Infrared Spectrosc..

[8] Dufour E., Sun D.-W. (2009). Principles of infrared spectroscopy. Infrared Spectroscopy for Food Quality Analysis and Control.

[9] Abbas O., Compère G., Larondelle Y., Pompeu D., Rogez H., Baeten V. (2017). Phenolic compound explorer: A mid-infrared spectroscopy database. Vib. Spectrosc..

[10] Mecozzi M., Sturchio E. (2017). Computer Assisted Examination of Infrared and Near Infrared Spectra to Assess Structural and Molecular Changes in Biological Samples Exposed to Pollutants: A Case of Study. J. Imaging.

[11] Clark C.J., McGlone V.A., Jordan R.B. (2003). Detection of Brownheart in ‘Braeburn’ apple by transmission NIR spectroscopy. Postharvest Biol. Technol..

[12] Fraser D.G., Jordan R.B., Künnemeyer R., McGlone V.A. (2003). Light distribution inside mandarin fruit during internal quality assessment by NIR spectroscopy. Postharvest Biol. Technol..

[13] Ncama K., Tesfay S.Z., Fawole O.A., Opara U.L., Magwaza L.S. (2018). Non-destructive prediction of ‘Marsh’ grapefruit susceptibility to postharvest rind pitting disorder using reflectance Vis/NIR spectroscopy. Sci. Hortic..

[14] Kumar S., Singh R., Dhanani T., Yahia E.M. (2017). Rapid Estimation of Bioactive Phytochemicals in Vegetables and Fruits Using Near Infrared Reflectance Spectroscopy. Fruit and Vegetable Phytochemicals.

[15] Yang Y., Xu-zhen C., Gui-xing R. (2011). Application of Near-Infrared Reflectance Spectroscopy to the Evaluation of D-chiro-lnositol, Vitexin, and Isovitexin Contents in Mung Bean. Agric. Sci. China.

[16] Caporaso N., Whitworth M.B., Fisk I.D. (2018). Near-Infrared spectroscopy and hyperspectral imaging for non-destructive quality assessment of cereal grains. Appl. Spectrosc. Rev..

[17] Wilson R.H., Tapp H.S. (1999). Mid-infrared spectroscopy for food analysis: Recent new applications and relevant developments in sample presentation methods. TrAC Trends Anal. Chem..

[18] Kawano S., Siesler H.W., Ozaki Y., Kawata S., Heise H.M. (2008). Sampling and sample presentation. Near-Infrared Spectroscopy: Principles, Instruments, Applications.

[19] Gautam R., Vanga S., Ariese F., Umapathy S. (2015). Review of multidimensional data processing approaches for Raman and infrared spectroscopy. EPJ Tech. Instrum..

[20] Rinnan Å. (2014). Pre-processing in vibrational spectroscopy—When, why and how. Anal. Methods.

[21] Schoot M., Kapper C., van Kollenburg G.H., Postma G.J., van Kessel G., Buydens L.M.C., Jansen J.J. (2020). Investigating the need for preprocessing of near-infrared spectroscopic data as a function of sample size. Chemom. Intell. Lab. Syst..

[22] Dotto A.C., Dalmolin R.S.D., ten Caten A., Grunwald S. (2018). A systematic study on the application of scatter-corrective and spectral-derivative preprocessing for multivariate prediction of soil organic carbon by Vis-NIR spectra. Geoderma.

[23] Mishra P., Biancolillo A., Roger J.M., Marini F., Rutledge D.N. (2020). New data preprocessing trends based on ensemble of multiple preprocessing techniques. TrAC Trends Anal. Chem..

[24] Lee L.C., Liong C.-Y., Jemain A.A. (2017). A contemporary review on Data Preprocessing (DP) practice strategy in ATR-FTIR spectrum. Chemom. Intell. Lab. Syst..

[25] Rodriguez-Otero J.L., Hermida M., Cepeda A. (1995). Determination of Fat, Protein, and Total Solids in Cheese by Near-Infrared Reflectance Spectroscopy. J. AOAC Int..

[26] Orman B.A., Schumann R.A. (1991). Comparison of near-infrared spectroscopy calibration methods for the prediction of protein, oil, and starch in maize grain. J. Agric. Food Chem..

[27] Terhoeven-Urselmans T., Schmidt H., Joergensen R.G., Ludwig B. (2008). Usefulness of near-infrared spectroscopy to determine biological and chemical soil properties: Importance of sample pre-treatment. Soil Biol. Biochem..

[28] Mehmood T., Ahmed B. (2016). The diversity in the applications of partial least squares: An overview. J. Chemom..

[29] Cobaleda-Velasco M., Almaraz-Abarca N., Alanis-Bañuelos R.E., Uribe-Soto J.N., González-Valdez L.S., Muñoz-Hernández G., Zaca-Morán O., Rojas-López M. (2018). Rapid determination of phenolics, flavonoids, and antioxidant properties of *Physalis ixocarpa* Brot. ex Hornem. and *Physalis angulata* L. by infrared spectroscopy and partial least squares. Anal. Lett..

[30] de Oliveira G.A., Bureau S., Renard C.M.-G.C., Pereira-Netto A.B., de Castilhos F. (2014). Comparison of NIRS approach for prediction of internal quality traits in three fruit species. Food Chem..

[31] Hu Y., Pan Z.J., Liao W., Li J., Gruget P., Kitts D.D., Lu X. (2016). Determination of antioxidant capacity and phenolic content of chocolate by attenuated total reflectance-Fourier transformed-infrared spectroscopy. Food Chem..

[32] Gabriëls S.H.E.J., Mishra P., Mensink M.G.J., Spoelstra P., Woltering E.J. (2020). Non-destructive measurement of internal browning in mangoes using visible and near-infrared spectroscopy supported by artificial neural network analysis. Postharvest Biol. Technol..

[33] Sharabiani V.R., Nazarloo A.S., Taghinezhad E. (2019). Prediction of Protein Content of Winter Wheat by Canopy of Near Infrared Spectroscopy (NIRS), Using Partial Least Squares Regression (PLSR) and Artificial Neural Network (ANN) Models. Yüzüncü Yıl Üniversitesi Tarım Bilim. Derg..

[34] Le B.T. (2020). Application of deep learning and near infrared spectroscopy in cereal analysis. Vib. Spectrosc..

[35] Rajalakshmi G., Gopal A. (2020). Performance evaluation of preprocessing techniques for near-infrared spectroscopy signals. Microprocess. Microsyst..

[36] Ludwig B., Murugan R., Parama V.R.R., Vohland M. (2019). Accuracy of Estimating Soil Properties with Mid-Infrared Spectroscopy: Implications of Different Chemometric Approaches and Software Packages Related to Calibration Sample Size. Soil Sci. Soc. Am. J..

[37] Ni W., Nørgaard L., Mørup M. (2014). Non-linear calibration models for near infrared spectroscopy. Anal. Chim. Acta.

[38] Urala N., Lähteenmäki L. (2007). Consumers’ changing attitudes towards functional foods. Food Qual. Prefer..

[39] Granato D., Nunes D.S., Barba F.J. (2017). An integrated strategy between food chemistry, biology, nutrition, pharmacology, and statistics in the development of functional foods: A proposal. Trends Food Sci. Technol..

[40] Johnson J.B., Walsh K.B., Mani J.S., Bhattarai S., Naiker M. More than Protein: The Potential for Rapid Assessment of Bioactive Compounds in Australian Crops. Proceedings of the Developing Northern Australia Conference.

[41] Netzel M., Fanning K., Netzel G., Zabaras D., Karagianis G., Treloar T., Russell D., Stanley R. (2012). Urinary excretion of antioxidants in healthy humans following Queen Garnet plum juice ingestion: A new plum variety rich in antioxidant compounds. J. Food Biochem..

[42] Santhakumar A.B., Kundur A.R., Fanning K., Netzel M., Stanley R., Singh I. (2015). Consumption of anthocyanin-rich Queen Garnet plum juice reduces platelet activation related thrombogenesis in healthy volunteers. J. Funct. Foods.

[43] Bochenek H.F., Santhakumar A.B., Francis N., Blanchard C.L., Chinkwo K.A. Anti-cancer effects of chickpea extracts. Proceedings of the 69th Australasian Grain Science Conference.

[44] Di Pasquale J., Adinolfi F., Capitanio F. (2011). Analysis of consumer attitudes and consumers’ willingness to pay for functional foods. Int. J. Food Syst. Dyn..

[45] Barreiro-Hurlé J., Colombo S., Cantos-Villar E. (2008). Is there a market for functional wines? Consumer preferences and willingness to pay for resveratrol-enriched red wine. Food Qual. Prefer..

[46] Hirogaki M. (2013). Estimating consumers’ willingness to pay for health food claims: A conjoint analysis. Int. J. Innov. Manag. Technol..

[47] Markosyan A., McCluskey J.J., Wahl T.I. (2009). Consumer Response to Information about a Functional Food Product: Apples Enriched with Antioxidants. Can. J. Agric. Econ. Rev. Can. D’agroeconomie.

[48] Miškolci S. (2014). Consumer preferences and willingness to pay for the health aspects of food. Acta Univ. Agric. Silvic. Mendel. Brun..

[49] López-Barrios L., Gutiérrez-Uribe J.A., Serna-Saldívar S.O. (2014). Bioactive peptides and hydrolysates from pulses and their potential use as functional ingredients. J. Food Sci..

[50] Vioque J., Alaiz M., Girón-Calle J. (2012). Nutritional and functional properties of Vicia faba protein isolates and related fractions. Food Chem..

[51] Zhang Z.-L., Zhou M.-L., Tang Y., Li F.-L., Tang Y.-X., Shao J.-R., Xue W.-T., Wu Y.-M. (2012). Bioactive compounds in functional buckwheat food. Food Res. Int..

[52] Boukid F., Folloni S., Sforza S., Vittadini E., Prandi B. (2018). Current Trends in Ancient Grains-Based Foodstuffs: Insights into Nutritional Aspects and Technological Applications. Compr. Rev. Food Sci. Food Saf..

[53] Cooper R. (2015). Re-discovering ancient wheat varieties as functional foods. J. Tradit. Complement. Med..

[54] Singh B., Singh J.P., Shevkani K., Singh N., Kaur A. (2017). Bioactive constituents in pulses and their health benefits. J. Food Sci. Technol..

[55] Fanning K., Edwards D., Netzel M., Stanley R., Netzel G., Russell D., Topp B. Increasing anthocyanin content in Queen Garnet plum and correlations with in-field measures. Proceedings of the X International Symposium on Plum and Prune Genetics, Breeding and Pomology.

[56] Xiang J., Zhang M., Apea-Bah F.B., Beta T. (2019). Hydroxycinnamic acid amide (HCAA) derivatives, flavonoid C-glycosides, phenolic acids and antioxidant properties of foxtail millet. Food Chem..

[57] de Lima Yamaguchi K.K., Pereira L.F.R., Lamarão C.V., Lima E.S., da Veiga-Junior V.F. (2015). Amazon acai: Chemistry and biological activities: A review. Food Chem..

[58] Guaadaoui A., Benaicha S., Elmajdoub N., Bellaoui M., Hamal A. (2014). What is a bioactive compound? A combined definition for a preliminary consensus. Int. J. Nutr. Food Sci..

[59] Biesalski H.-K., Dragsted L.O., Elmadfa I., Grossklaus R., Müller M., Schrenk D., Walter P., Weber P. (2009). Bioactive compounds: Definition and assessment of activity. Nutrition.

[60] Kalra E.K. (2003). Nutraceutical—Definition and introduction. AAPS PharmSci.

[61] Hamzalıoğlu A., Gökmen V., Gökmen V. (2016). Chapter 18—Interaction between Bioactive Carbonyl Compounds and Asparagine and Impact on Acrylamide. Acrylamide in Food.

[62] Mani J.S., Johnson J.B., Hosking H., Ashwath N., Walsh K.B., Neilsen P.M., Broszczak D.A., Naiker M. (2021). Antioxidative and therapeutic potential of selected Australian plants: A review. J. Ethnopharmacol..

[63] Câmara J.S., Albuquerque B.R., Aguiar J., Corrêa R.C.G., Gonçalves J.L., Granato D., Pereira J.A.M., Barros L., Ferreira I.C.F.R. (2021). Food Bioactive Compounds and Emerging Techniques for Their Extraction: Polyphenols as a Case Study. Foods.

[64] Pellegrini N., Vitaglione P., Granato D., Fogliano V. (2020). Twenty-five years of total antioxidant capacity measurement of foods and biological fluids: Merits and limitations. J. Sci. Food Agric..

[65] Pompella A., Sies H., Wacker R., Brouns F., Grune T., Biesalski H.K., Frank J. (2014). The use of total antioxidant capacity as surrogate marker for food quality and its effect on health is to be discouraged. Nutrition.

[66] Fraga C.G., Oteiza P.I., Galleano M. (2014). In vitro measurements and interpretation of total antioxidant capacity. Biochim. Biophys. Acta (BBA) Gen. Subj..

[67] Hermsdorff H.H.M., Puchau B., Volp A.C.P., Barbosa K.B.F., Bressan J., Zulet M.Á., Martínez J.A. (2011). Dietary total antioxidant capacity is inversely related to central adiposity as well as to metabolic and oxidative stress markers in healthy young adults. Nutr. Metab..

[68] Wang Y., Yang M., Lee S.-G., Davis C.G., Koo S.I., Fernandez M.L., Volek J.S., Chun O.K. (2014). Diets high in total antioxidant capacity improve risk biomarkers of cardiovascular disease: A 9-month observational study among overweight/obese postmenopausal women. Eur. J. Nutr..

[69] Kobayashi S., Murakami K., Sasaki S., Uenishi K., Yamasaki M., Hayabuchi H., Goda T., Oka J., Baba K., Ohki K. (2012). Dietary total antioxidant capacity from different assays in relation to serum C-reactive protein among young Japanese women. Nutr. J..

[70] Detopoulou P., Panagiotakos D.B., Chrysohoou C., Fragopoulou E., Nomikos T., Antonopoulou S., Pitsavos C., Stefanadis C. (2010). Dietary antioxidant capacity and concentration of adiponectin in apparently healthy adults: The ATTICA study. Eur. J. Clin. Nutr..

[71] Agudo A., Cabrera L., Amiano P., Ardanaz E., Barricarte A., Berenguer T., Chirlaque M.D., Dorronsoro M., Jakszyn P., Larrañaga N. (2007). Fruit and vegetable intakes, dietary antioxidant nutrients, and total mortality in Spanish adults: Findings from the Spanish cohort of the European Prospective Investigation into Cancer and Nutrition (EPIC-Spain). Am. J. Clin. Nutr..

[72] Bastide N., Dartois L., Dyevre V., Dossus L., Fagherazzi G., Serafini M., Boutron-Ruault M.-C. (2017). Dietary antioxidant capacity and all-cause and cause-specific mortality in the E3N/EPIC cohort study. Eur. J. Nutr..

[73] Rautiainen S., Levitan E.B., Orsini N., Åkesson A., Morgenstern R., Mittleman M.A., Wolk A. (2012). Total Antioxidant Capacity from Diet and Risk of Myocardial Infarction: A Prospective Cohort of Women. Am. J. Med..

[74] Rautiainen S., Levitan E.B., Mittleman M.A., Wolk A. (2013). Total Antioxidant Capacity of Diet and Risk of Heart Failure: A Population-based Prospective Cohort of Women. Am. J. Med..

[75] Rautiainen S., Larsson S., Virtamo J., Wolk A. (2012). Total Antioxidant Capacity of Diet and Risk of Stroke. Stroke.

[76] Del Rio D., Agnoli C., Pellegrini N., Krogh V., Brighenti F., Mazzeo T., Masala G., Bendinelli B., Berrino F., Sieri S. (2010). Total Antioxidant Capacity of the Diet Is Associated with Lower Risk of Ischemic Stroke in a Large Italian Cohort. J. Nutr..

[77] Colarusso L., Serafini M., Lagerros Y.T., Nyren O., La Vecchia C., Rossi M., Ye W., Tavani A., Adami H.-O., Grotta A. (2017). Dietary antioxidant capacity and risk for stroke in a prospective cohort study of Swedish men and women. Nutrition.

[78] Kukula-Koch W., Koch W., Czernicka L., Głowniak K., Asakawa Y., Umeyama A., Marzec Z., Kuzuhara T. (2018). MAO-A Inhibitory Potential of Terpene Constituents from Ginger Rhizomes—A Bioactivity Guided Fractionation. Molecules.

[79] da Silva A.P.G., Spricigo P.C., Purgatto E., de Alencar S.M., Jacomino A.P. (2019). *Plinia trunciflora* and *Plinia cauliflora*: Two species rich in bioactive compounds, terpenes, and minerals. J. Food Meas. Charact..

[80] Xiang J., Apea-Bah F.B., Ndolo V.U., Katundu M.C., Beta T. (2019). Profile of phenolic compounds and antioxidant activity of finger millet varieties. Food Chem..

[81] Netzel M., Netzel G., Tian Q., Schwartz S., Konczak I. (2006). Sources of Antioxidant Activity in Australian Native Fruits. Identification and Quantification of Anthocyanins. J. Agric. Food Chem..

[82] Baysal I., Ekizoglu M., Ertas A., Temiz B., Agalar H.G., Yabanoglu-Ciftci S., Temel H., Ucar G., Turkmenoglu F.P. (2021). Identification of Phenolic Compounds by LC-MS/MS and Evaluation of Bioactive Properties of Two Edible Halophytes: *Limonium effusum* and *L. sinuatum*. Molecules.

[83] White R. (1990). Chromatography/Fourier Transform Infrared Spectroscopy and Its Applications.

[84] Cordella C., Moussa I., Martel A.-C., Sbirrazzuoli N., Lizzani-Cuvelier L. (2002). Recent Developments in Food Characterization and Adulteration Detection:  Technique-Oriented Perspectives. J. Agric. Food Chem..

[85] Salerno T.M.G., Donato P., Frison G., Zamengo L., Mondello L. (2020). Gas Chromatography—Fourier Transform Infrared Spectroscopy for Unambiguous Determination of Illicit Drugs: A Proof of Concept. Front. Chem..

[86] Frison G., Zancanaro F., Frasson S., Quadretti L., Agnati M., Vlassich F., Gagliardi G., Salerno T.M.G., Donato P., Mondello L. (2021). Analytical Characterization of 3-MeO-PCP and 3-MMC in Seized Products and Biosamples: The Role of LC-HRAM-Orbitrap-MS and Solid Deposition GC-FTIR. Front. Chem..

[87] Salerno T.M.G., Coppolino C., Donato P., Mondello L. (2022). The online coupling of liquid chromatography to Fourier transform infrared spectroscopy using a solute-deposition interface: A proof of concept. Anal. Bioanal. Chem..

[88] Dos Santos W.N.L., da Silva Sauthier M.C., dos Santos A.M.P., de Andrade Santana D., Azevedo R.S.A., da Cruz Caldas J. (2017). Simultaneous determination of 13 phenolic bioactive compounds in guava (*Psidium guajava* L.) by HPLC-PAD with evaluation using PCA and Neural Network Analysis (NNA). Microchem. J..

[89] Johnson J., Collins T., Walsh K., Naiker M. (2020). Solvent extractions and spectrophotometric protocols for measuring the total anthocyanin, phenols and antioxidant content in plums. Chem. Pap..

[90] Johnson J., Mani J., Ashwath N., Naiker M. (2020). Potential for Fourier transform infrared (FTIR) spectroscopy toward predicting antioxidant and phenolic contents in powdered plant matrices. Spectrochim. Acta Part A Mol. Biomol. Spectrosc..

[91] Mahesar S.A., Lucarini M., Durazzo A., Santini A., Lampe A.I., Kiefer J. (2019). Application of Infrared Spectroscopy for Functional Compounds Evaluation in Olive Oil: A Current Snapshot. J. Spectrosc..

[92] Aleixandre-Tudo J.L., Nieuwoudt H., Aleixandre J.L., du Toit W. (2018). Chemometric compositional analysis of phenolic compounds in fermenting samples and wines using different infrared spectroscopy techniques. Talanta.

[93] Cozzolino D. (2015). Infrared Spectroscopy as a Versatile Analytical Tool for the Quantitative Determination of Antioxidants in Agricultural Products, Foods and Plants. Antioxidants.

[94] Lu X., Rasco B.A. (2012). Determination of Antioxidant Content and Antioxidant Activity in Foods using Infrared Spectroscopy and Chemometrics: A Review. Crit. Rev. Food Sci. Nutr..

[95] Ignat I., Volf I., Popa V.I. (2011). A critical review of methods for characterisation of polyphenolic compounds in fruits and vegetables. Food Chem..

[96] McGoverin C.M., Weeranantanaphan J., Downey G., Manley M. (2010). Review: The Application of near Infrared Spectroscopy to the Measurement of Bioactive Compounds in Food Commodities. J. Near Infrared Spectrosc..

[97] Pallone J.A.L., dos Santos Caramês E.T., Alamar P.D. (2018). Green analytical chemistry applied in food analysis: Alternative techniques. Curr. Opin. Food Sci..

[98] Júnior L.C.C., de Almeida Teixeira G.H., Nardini V., Walsh K.B. (2016). Quality evaluation of intact açaí and juçara fruit by means of near infrared spectroscopy. Postharvest Biol. Technol..

[99] Gardana C., Scialpi A., Fachechi C., Simonetti P. (2018). Near-Infrared Spectroscopy and Chemometrics for the Routine Detection of Bilberry Extract Adulteration and Quantitative Determination of the Anthocyanins. J. Spectrosc..

[100] Toledo-Martín E.M., García-García M.D.C., Font R., Moreno-Rojas J.M., Salinas-Navarro M., Gómez P., del Río-Celestino M. (2018). Quantification of Total Phenolic and Carotenoid Content in Blackberries (*Rubus fructicosus* L.) Using Near Infrared Spectroscopy (NIRS) and Multivariate Analysis. Molecules.

[101] Tzanova M., Atanassova S., Atanasov V., Grozeva N. (2020). Content of Polyphenolic Compounds and Antioxidant Potential of Some Bulgarian Red Grape Varieties and Red Wines, Determined by HPLC, UV, and NIR Spectroscopy. Agriculture.

[102] Xiao H., Li A., Li M., Sun Y., Tu K., Wang S., Pan L. (2018). Quality assessment and discrimination of intact white and red grapes from *Vitis vinifera* L. at five ripening stages by visible and near-infrared spectroscopy. Sci. Hortic..

[103] Diago M.P., Fernández-Novales J., Fernandes A.M., Melo-Pinto P., Tardaguila J. (2016). Use of Visible and Short-Wave Near-Infrared Hyperspectral Imaging To Fingerprint Anthocyanins in Intact Grape Berries. J. Agric. Food Chem..

[104] Martínez-Sandoval J.R., Nogales-Bueno J., Rodríguez-Pulido F.J., Hernández-Hierro J.M., Segovia-Quintero M.A., Martínez-Rosas M.E., Heredia F.J. (2016). Screening of anthocyanins in single red grapes using a non-destructive method based on the near infrared hyperspectral technology and chemometrics. J. Sci. Food Agric..

[105] Jara-Palacios M.J., Rodríguez-Pulido F.J., Hernanz D., Escudero-Gilete M.L., Heredia F.J. (2016). Determination of phenolic substances of seeds, skins and stems from white grape marc by near-infrared hyperspectral imaging. Aust. J. Grape Wine Res..

[106] Zhang N., Liu X., Jin X., Li C., Wu X., Yang S., Ning J., Yanne P. (2017). Determination of total iron-reactive phenolics, anthocyanins and tannins in wine grapes of skins and seeds based on near-infrared hyperspectral imaging. Food Chem..

[107] Alamar P.D., Caramês E.T.S., Poppi R.J., Pallone J.A.L. (2016). Quality evaluation of frozen guava and yellow passion fruit pulps by NIR spectroscopy and chemometrics. Food Res. Int..

[108] Arslan M., Xiaobo Z., Shi J., Tahir H.E., Zareef M., Rakha A., Bilal M. (2020). In situ prediction of phenolic compounds in puff dried *Ziziphus jujuba* Mill. using hand-held spectral analytical system. Food Chem..

[109] Cozzolino D., Phan A.D.T., Netzel M.E., Smyth H., Sultanbawa Y. (2020). The use of vibrational spectroscopy to predict vitamin C in Kakadu plum powders (*Terminalia ferdinandiana* Exell, Combretaceae). J. Sci. Food Agric..

[110] Rodríguez-Pulido F.J., Gil-Vicente M., Gordillo B., Heredia F.J., González-Miret M.L. (2017). Measurement of ripening of raspberries (*Rubus idaeus* L) by near infrared and colorimetric imaging techniques. J. Food Sci. Technol..

[111] Alenazi M.M., Shafiq M., Alsadon A.A., Alhelal I.M., Alhamdan A.M., Solieman T.H.I., Ibrahim A.A., Shady M.R., Saad M.A.O. (2020). Non-destructive assessment of flesh firmness and dietary antioxidants of greenhouse-grown tomato (*Solanum lycopersicum* L.) at different fruit maturity stages. Saudi J. Biol. Sci..

[112] Tilahun S., Park D.S., Seo M.H., Hwang I.G., Kim S.H., Choi H.R., Jeong C.S. (2018). Prediction of lycopene and β-carotene in tomatoes by portable chroma-meter and VIS/NIR spectra. Postharvest Biol. Technol..

[113] Ding X., Guo Y., Ni Y., Kokot S. (2016). A novel NIR spectroscopic method for rapid analyses of lycopene, total acid, sugar, phenols and antioxidant activity in dehydrated tomato samples. Vib. Spectrosc..

[114] Viegas T.R., Mata A.L.M.L., Duarte M.M.L., Lima K.M.G. (2016). Determination of quality attributes in wax jambu fruit using NIRS and PLS. Food Chem..

[115] Rady A.M., Sugiharto S., Adedeji A.A. (2018). Evaluation of carrot quality using visible near infrared spectroscopy and multivariate analysis. J. Food Res..

[116] de Oliveira I.R.N., Roque J.V., Maia M.P., Stringheta P.C., Teófilo R.F. (2018). New strategy for determination of anthocyanins, polyphenols and antioxidant capacity of Brassica oleracea liquid extract using infrared spectroscopies and multivariate regression. Spectrochim. Acta Part A Mol. Biomol. Spectrosc..

[117] López-Maestresalas A., Pérez C., Tierno R., Arazuri S., De Galarreta J.I.R., Jarén C. (2017). Prediction of main potato compounds by NIRS. Chem. Eng. Trans..

[118] Tschannerl J., Ren J., Jack F., Krause J., Zhao H., Huang W., Marshall S. (2019). Potential of UV and SWIR hyperspectral imaging for determination of levels of phenolic flavour compounds in peated barley malt. Food Chem..

[119] Wiedemair V., Huck C.W. (2018). Evaluation of the performance of three hand-held near-infrared spectrometer through investigation of total antioxidant capacity in gluten-free grains. Talanta.

[120] Carbas B., Machado N., Oppolzer D., Queiroz M., Brites C., Rosa E.A.S., Barros A.I.R.N.A. (2020). Prediction of Phytochemical Composition, In Vitro Antioxidant Activity and Individual Phenolic Compounds of Common Beans Using MIR and NIR Spectroscopy. Food Bioprocess Technol..

[121] Meenu M., Kamboj U., Sharma A., Guha P., Mishra S. (2016). Green method for determination of phenolic compounds in mung bean (*Vigna radiata* L.) based on near-infrared spectroscopy and chemometrics. Int. J. Food Sci. Technol..

[122] Macavilca E.A., Condezo-Hoyos L. (2020). Assessment of total antioxidant capacity of altiplano colored quinoa (*Chenopodium quinoa* willd) by visible and near-infrared diffuse reflectance spectroscopy and chemometrics. LWT.

[123] Amanah H.Z., Joshi R., Masithoh R.E., Choung M.-G., Kim K.-H., Kim G., Cho B.-K. (2020). Nondestructive measurement of anthocyanin in intact soybean seed using Fourier Transform Near-Infrared (FT-NIR) and Fourier Transform Infrared (FT-IR) spectroscopy. Infrared Phys. Technol..

[124] Cayuela J.A., García J.F. (2018). Nondestructive measurement of squalene in olive oil by near infrared spectroscopy. LWT.

[125] Cayuela J.A., García J.F. (2017). Sorting olive oil based on alpha-tocopherol and total tocopherol content using near-infra-red spectroscopy (NIRS) analysis. J. Food Eng..

[126] Mora-Ruiz M.E., Reboredo-Rodríguez P., Salvador M.D., González-Barreiro C., Cancho-Grande B., Simal-Gándara J., Fregapane G. (2017). Assessment of polar phenolic compounds of virgin olive oil by NIR and mid-IR spectroscopy and their impact on quality. Eur. J. Lipid Sci. Technol..

[127] Trapani S., Migliorini M., Cecchi L., Giovenzana V., Beghi R., Canuti V., Fia G., Zanoni B. (2017). Feasibility of filter-based NIR spectroscopy for the routine measurement of olive oil fruit ripening indices. Eur. J. Lipid Sci. Technol..

[128] Park J.-R., Kang H.-H., Cho J.-K., Moon K.-D., Kim Y.-J. (2020). Feasibility of rapid piperine quantification in whole and black pepper using near infrared spectroscopy and chemometrics. J. Food Sci..

[129] Wang Y.-J., Li T.-H., Li L.-Q., Ning J.-M., Zhang Z.-Z. (2021). Evaluating taste-related attributes of black tea by micro-NIRS. J. Food Eng..

[130] Zareef M., Chen Q., Ouyang Q., Arslan M., Hassan M.M., Ahmad W., Viswadevarayalu A., Wang P., Ancheng W. (2019). Rapid screening of phenolic compounds in congou black tea (*Camellia sinensis*) during in vitro fermentation process using portable spectral analytical system coupled chemometrics. J. Food Process. Preserv..

[131] Hernández-Hernández C., Fernández-Cabanás V.M., Rodríguez-Gutiérrez G., Bermúdez-Oria A., Morales-Sillero A. (2021). Viability of near infrared spectroscopy for a rapid analysis of the bioactive compounds in intact cocoa bean husk. Food Control.

[132] Sunoj S., Igathinathane C., Visvanathan R. (2016). Nondestructive determination of cocoa bean quality using FT-NIR spectroscopy. Comput. Electron. Agric..

[133] Nogales-Bueno J., Baca-Bocanegra B., Romero-Molina L., Martínez-López A., Rato A.E., Heredia F.J., Hernández-Hierro J.M., Escudero-Gilete M.L., González-Miret M.L. (2020). Control of the extractable content of bioactive compounds in coffee beans by near infrared hyperspectral imaging. LWT.

[134] Yan H., Li P.-H., Zhou G.-S., Wang Y.-J., Bao B.-H., Wu Q.-N., Huang S.-L. (2021). Rapid and practical qualitative and quantitative evaluation of non-fumigated ginger and sulfur-fumigated ginger via Fourier-transform infrared spectroscopy and chemometric methods. Food Chem..

[135] Caramês E.T.S., Alamar P.D., Poppi R.J., Pallone J.A.L. (2017). Quality control of cashew apple and guava nectar by near infrared spectroscopy. J. Food Compos. Anal..

[136] Shan J., Wang X., Han S., Kondo N. (2017). Application of Curve Fitting and Wavelength Selection Methods for Determination of Chlorogenic Acid Concentration in Coffee Aqueous Solution by Vis/NIR Spectroscopy. Food Anal. Methods.

[137] Caramês E.T.S., Alamar P.D., Poppi R.J., Pallone J.A.L. (2017). Rapid Assessment of Total Phenolic and Anthocyanin Contents in Grape Juice Using Infrared Spectroscopy and Multivariate Calibration. Food Anal. Methods.

[138] Santana M.C.D., Ferreira M.M.C., Pallone J.A.L. (2020). Control of ascorbic acid in fortified powdered soft drinks using near-infrared spectroscopy (NIRS) and multivariate analysis. J. Food Sci. Technol..

[139] Tahir H.E., Xiaobo Z., Tinting S., Jiyong S., Mariod A.A. (2016). Near-Infrared (NIR) Spectroscopy for Rapid Measurement of Antioxidant Properties and Discrimination of Sudanese Honeys from Different Botanical Origin. Food Anal. Methods.

[140] Betances-Salcedo E., Revilla I., Vivar-Quintana A.M., González-Martín M.I. (2017). Flavonoid and Antioxidant Capacity of Propolis Prediction Using Near Infrared Spectroscopy. Sensors.

[141] Kokalj Ladan M., Straus J., Tavčar Benković E., Kreft S. (2017). FT-IR-based method for rutin, quercetin and quercitrin quantification in different buckwheat (*Fagopyrum*) species. Sci. Rep..

[142] Uncu O., Ozen B., Tokatli F. (2019). Use of FTIR and UV–visible spectroscopy in determination of chemical characteristics of olive oils. Talanta.

[143] Hirri A., Bassbasi M., Souhassou S., Kzaiber F., Oussama A. (2016). Prediction of Polyphenol Fraction in Virgin Olive Oil Using Mid-Infrared Attenuated Total Reflectance Attenuated Total Reflectance Accessory–Mid-Infrared Coupled with Partial Least Squares Regression. Int. J. Food Prop..

[144] Carvalho D.G., Ranzan L., Trierweiler L.F., Trierweiler J.O. (2020). Determination of the concentration of total phenolic compounds in aged cachaça using two-dimensional fluorescence and mid-infrared spectroscopy. Food Chem..

[145] Ristic R., Cozzolino D., Jeffery D.W., Gambetta J.M., Bastian S.E.P. (2016). Prediction of Phenolic Composition of Shiraz Wines Using Attenuated Total Reflectance Mid-Infrared (ATR-MIR) Spectroscopy. Am. J. Enol. Vitic..

[146] Canal C., Ozen B. (2017). Monitoring of Wine Process and Prediction of Its Parameters with Mid-Infrared Spectroscopy. J. Food Process Eng..

[147] Sen I., Ozturk B., Tokatli F., Ozen B. (2016). Combination of visible and mid-infrared spectra for the prediction of chemical parameters of wines. Talanta.

[148] Tahir H.E., Xiaobo Z., Zhihua L., Jiyong S., Zhai X., Wang S., Mariod A.A. (2017). Rapid prediction of phenolic compounds and antioxidant activity of Sudanese honey using Raman and Fourier transform infrared (FT-IR) spectroscopy. Food Chem..

[149] Beć K.B., Grabska J., Huck C.W. (2021). NIR spectroscopy of natural medicines supported by novel instrumentation and methods for data analysis and interpretation. J. Pharm. Biomed. Anal..

[150] Anderson N.T., Walsh K.B., Flynn J.R., Walsh J.P. (2021). Achieving robustness across season, location and cultivar for a NIRS model for intact mango fruit dry matter content. II. Local PLS and nonlinear models. Postharvest Biol. Technol..

[151] Johnson J.B., Broszczak D.A., Mani J.S., Anesi J., Naiker M. (2022). A cut above the rest: Oxidative stress in chronic wounds and the potential role of polyphenols as therapeutics. J. Pharm. Pharmacol..

[152] Rasouli H., Farzaei M.H., Khodarahmi R. (2017). Polyphenols and their benefits: A review. Int. J. Food Prop..

[153] Alves-Santos A.M., Sugizaki C.S.A., Lima G.C., Naves M.M.V. (2020). Prebiotic effect of dietary polyphenols: A systematic review. J. Funct. Foods.

[154] Koch W. (2019). Dietary Polyphenols—Important Non-Nutrients in the Prevention of Chronic Noncommunicable Diseases. A Systematic Review. Nutrients.

[155] Cassidy L., Fernandez F., Johnson J.B., Naiker M., Owoola A.G., Broszczak D.A. (2020). Oxidative Stress in Alzheimer’s Disease: A Review on Emergent Natural Polyphenolic Therapeutics. Complement. Ther. Med..

[156] Rasines-Perea Z., Teissedre P.-L. (2017). Grape Polyphenols’ Effects in Human Cardiovascular Diseases and Diabetes. Molecules.

[157] Arbeláez L.F.G., Pardo A.C., Fantinelli J.C., Schinella G.R., Mosca S.M., Ríos J.-L. (2018). Cardioprotection and natural polyphenols: An update of clinical and experimental studies. Food Funct..

[158] Costa C., Tsatsakis A., Mamoulakis C., Teodoro M., Briguglio G., Caruso E., Tsoukalas D., Margina D., Dardiotis E., Kouretas D. (2017). Current evidence on the effect of dietary polyphenols intake on chronic diseases. Food Chem. Toxicol..

[159] Sanches-Silva A., Testai L., Nabavi S.F., Battino M., Devi K.P., Tejada S., Sureda A., Xu S., Yousefi B., Majidinia M. (2020). Therapeutic potential of polyphenols in cardiovascular diseases: Regulation of mTOR signaling pathway. Pharmacol. Res..

[160] Ed Nignpense B., Chinkwo K.A., Blanchard C.L., Santhakumar A.B. (2020). Polyphenols: Modulators of Platelet Function and Platelet Microparticle Generation?. Int. J. Mol. Sci..

[161] Ferrer-Gallego R., Rodríguez-Pulido F.J., Toci A.T., García-Estevez I. (2020). Phenolic Composition, Quality and Authenticity of Grapes and Wines by Vibrational Spectroscopy. Food Rev. Int..

[162] Martín-Tornero E., de Jorge Páscoa R.N.M., Espinosa-Mansilla A., Martín-Merás I.D., Lopes J.A. (2020). Comparative quantification of chlorophyll and polyphenol levels in grapevine leaves sampled from different geographical locations. Sci. Rep..

[163] Lu Y., Saeys W., Kim M., Peng Y., Lu R. (2020). Hyperspectral imaging technology for quality and safety evaluation of horticultural products: A review and celebration of the past 20-year progress. Postharvest Biol. Technol..

[164] Caporaso N., ElMasry G., Gou P., Galanakis C.M. (2021). Chapter 13—Hyperspectral imaging techniques for noncontact sensing of food quality. Innovative Food Analysis.

[165] Wang B., Sun J., Xia L., Liu J., Wang Z., Li P., Guo Y., Sun X. (2021). The Applications of Hyperspectral Imaging Technology for Agricultural Products Quality Analysis: A Review. Food Rev. Int..

[166] Khan A., Munir M.T., Yu W., Young B.R. (2020). A Review Towards Hyperspectral Imaging for Real-Time Quality Control of Food Products with an Illustrative Case Study of Milk Powder Production. Food Bioprocess Technol..

[167] Temiz H.T., Ulaş B. (2021). A Review of Recent Studies Employing Hyperspectral Imaging for the Determination of Food Adulteration. Photochem.

[168] Feng L., Wu B., Zhu S., He Y., Zhang C. (2021). Application of Visible/Infrared Spectroscopy and Hyperspectral Imaging with Machine Learning Techniques for Identifying Food Varieties and Geographical Origins. Front. Nutr..

[169] Kiani S., van Ruth S.M., Minaei S., Ghasemi-Varnamkhasti M. (2018). Hyperspectral imaging, a non-destructive technique in medicinal and aromatic plant products industry: Current status and potential future applications. Comput. Electron. Agric..

[170] Torres I., Sánchez M.-T., Garrido-Varo A., Pérez-Marín D. (2020). New Generation NIRS Sensors for Quality and Safety Assurance in Summer Squashes along the Food Supply Chain.

[171] Agbonkonkon N., Wojciechowski G., Abbott D.A., Gaucher S.P., Yim D.R., Thompson A.W., Leavell M.D. (2021). Faster, reduced cost calibration method development methods for the analysis of fermentation product using near-infrared spectroscopy (NIRS). J. Ind. Microbiol. Biotechnol..

